# Separation and Purification of Hydrocarbons with Porous Materials

**DOI:** 10.1002/anie.202104318

**Published:** 2021-05-06

**Authors:** Yaqi Wu, Bert M Weckhuysen

**Affiliations:** ^1^ Inorganic Chemistry and Catalysis Debye Institute for Nanomaterials Science Utrecht University Universiteitsweg 99 3584 CG Utrecht The Netherlands

**Keywords:** hydrocarbon separation, metal–organic frameworks, porous organic frameworks, porous materials, zeolites

## Abstract

This Minireview focuses on the developments of the adsorptive separation of methane/nitrogen, ethene/ethane, propene/propane mixtures as well as on the separation of C_8_ aromatics (i.e. xylene isomers) with a wide variety of materials, including carbonaceous materials, zeolites, metal–organic frameworks, and porous organic frameworks. Some recent important developments for these adsorptive separations are also highlighted. The advantages and disadvantages of each material category are discussed and guidelines for the design of improved materials are proposed. Furthermore, challenges and future developments of each material type and separation processes are discussed.

## Graphical abstract

This Minireview focuses on the developments of adsorbents for the separation and purification of hydrocarbons from, for example, methane/nitrogen, ethene/ethane, and propene/propane mixtures as well as for the separation of C_8_ aromatics (i.e. xylene isomers).

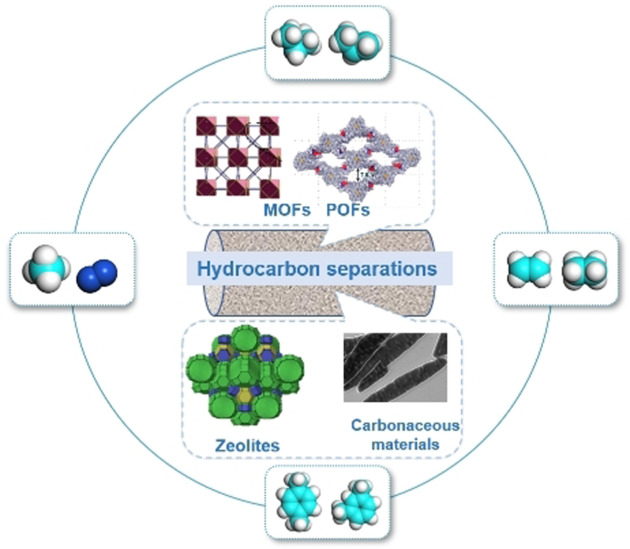

## 1 Introduction

Hydrocarbons play a significant role in the global energy structure as well as in the chemical and petrochemical industries. They are not only important fuels, such as gasoline, diesel and kerosene, but also vital feedstocks for the production of many of our chemical products and materials, including pharmaceuticals, coatings, and plastics.^[1]^ However, hydrocarbons from crude oil or natural gas always exist as mixtures, which need to be separated and purified to a single component for the further production of, for example, plastics.^[2]^ Separation of hydrocarbons having comparable sizes and molecular structures, and consequently similar physicochemical properties, is still a challenging endeavor. Adsorbent‐based separation technologies are potential alternatives to the current industrially used cryogenic distillation.^[3]^ In this Minireview, we mainly focus on the separation and purification of hydrocarbons from methane and olefins (i.e. ethene and propene) to C_8_ aromatics (i.e. xylene isomers).

Natural gas is becoming more and more important in the global energy structure. It is reported that in 2018, global natural gas consumption grew at a rate of 5.3 %, one of the strongest rates of growth since 1984.^[4]^ With the increasing standards of living and related consumption, the global demand for natural gas has also risen sharply. The primary constituent of natural gas is methane, while it also contains a mixture of impurities, including C_2+_ hydrocarbons such as ethane and propane, as well as N_2_, CO_2_, water, and hydrogen sulfide or other sulfur compounds.^[5]^ All raw natural gases containing these contaminants require some treatment to meet pipeline specifications, typically >90 % methane.^[6]^ Furthermore, the exploitation of unconventional sources of natural gases, like shale gas, coalbed methane, methane from anaerobic wastewater treatment plants, and landfill gas, has greatly increased the accessible reserves of natural gas and these sources have become important in filling the gap between demand and supply. In general, water, N_2_, CO_2_, and sulfur are also typical impurities found in those unconventional natural gases. To improve the purity and upgrade the energy content of the natural gases, these contaminants need to be removed. Among them, the very similar physicochemical properties of CH_4_ and N_2_ makes CH_4_/N_2_ separation one of the most challenging and key separations for natural gas utilization.

Olefin/paraffin separation and the separation of xylene isomers are listed among the “seven chemical separations to change the world.” ^[1c]^ Ethene and propene are critical petrochemical feedstocks, and the starting chemicals for the most widely produced synthetic plastics worldwide, namely polyethylene (PE) and polypropylene (PP). Usually, ethene and propene are produced by the steam cracking of hydrocarbons ranging from ethane to vacuum gas oils (VGOs), in which many other hydrocarbons, such as ethane and propane, also co‐exist with ethene and propene.^[7]^ Because the production of PE and PP requires polymer‐grade (>99.5 %) ethene and propene, removal of ethane and propane are essential. Currently, ethene/ethane and propene/propane separation rely on the energy‐intensive cryogenic distillation, which is performed in large columns containing over 100 trays.^[8]^ The annual energy consumption in ethene and propene purification alone accounts for 0.3 % of the global energy use.^[1c]^ Adsorption‐based separation is believed to be an energy‐ and cost‐efficient alternative technology to accomplish this highly energy‐consuming process.

C_8_ aromatics, consisting of the three xylene isomers, *para*‐xylene (PX), *ortho*‐xylene (OX), and *meta*‐xylene (MX), and ethylbenzene (EB), are raw materials for the synthesis of many important chemical intermediates. PX, the starting material for the synthesis of terephthalic acid, is the most valuable commodity among C_8_ aromatics, because terephthalic acid is the key precursor for production of polymers such as polyethylene terephthalate (PET), and polyester. MX is oxidized to synthesize isophthalic acid, which can be used as a co‐monomer in the production of PET‐based resins blends. OX is basically used in the production of phthalic anhydride. EB is utilized in polystyrene (PS) production after undergoing a catalytic dehydrogenation to styrene. C_8_ aromatics are mainly produced by the catalytic reforming of crude oil, gasoline pyrolysis, and toluene disproportionation, which always generate a mixture that must be further separated.^[9]^ Owing to the similar boiling points, melting points, and structures of the C_8_ aromatics, separation of these isomers is expensive and very energy intensive.^[10]^ Currently, industrial separation of xylene isomers is mainly accomplished by crystallization and selective adsorption on zeolites. However, the development of adsorbents with higher efficiency is important and great efforts have been made worldwide.

Owing to the tremendous prospects of adsorptive separation technology, a vast variety of porous materials have been explored for the separation and purification of hydrocarbon mixtures. Porous materials, such as activated carbons, carbon molecular sieves, zeolites, activated aluminas, silica gels, polymer resins, metal–organic frameworks (MOFs), metal organic cages, and porous organic frameworks (POFs), have been extensively studied for adsorptive separations.[^3c^, ^11^] Porous carbons have been among the most investigated porous adsorbents with low cost, high specific surface areas, and high stability. It also has been used, for example, for CH_4_/N_2_ separation, ethene/ethane separation, and propene/propane separation. Earlier studies mainly focused on the methods like loading other chemicals into porous carbons to promote the separation performance.[^11a^, ^12^] In recent years, porous carbons derived from biomass have also attracted great attention,^[13]^ while some heteroatom‐doped porous carbon materials have also received considerable attention for hydrocarbon separations.^[14]^

The discovery of natural zeolites and the development of synthetic zeolites greatly broadened the available range of adsorbents and represents one of the major breakthroughs in gas adsorption and separation.^[15]^ So far, the number of zeolite framework types officially registered by the International Zeolite Association (IZA) is more than 250. With the great availability of zeolite framework structures, high stability, easy synthesis process, and low cost, zeolites have been broadly used for catalysis, ion exchange, and adsorption‐separation in the chemical industry.^[16]^ Noteworthy developments include improved adsorbents for xylene separation (in the so‐called Heavy Parex Process) and the development of Li^+^‐exchanged low silica zeolite X as the adsorbent of choice for the pressure swing‐adsorption (PSA) process to separate oxygen, nitrogen, and carbon dioxide from air.^[17]^ Zeolite research is ongoing with the possibility of preparing them in a wide range of chemical compositions and with a wide variety of framework structures. For hydrocarbon separations, both traditional zeolites, often after proper modifications, and newly synthesized zeolites have been developed as useful adsorbents.

In the past decades, the emergence of MOFs and POFs has brought new life to the field of hydrocarbon separation and purification. MOFs are well‐defined microporous crystalline materials, which consist of inorganic nodes (i.e. metal ions and their clusters) and organic linkers.^[18]^ Given the broad range of inorganic nodes and organic linkers, there is currently a large family of synthetic MOFs available. Due to the inherent diversity, the tunable pore geometry, and easy functionalization, MOFs exhibit great potential for the separation of hydrocarbon mixtures by various separation mechanisms.[^2b^, ^2c^, ^11f^] Along with MOFs, later discovered POFs also exhibit exceptional porosity and higher chemical stability and have gained great attention for gas storage, separation, catalysis, and electronics applications.^[19]^ POFs assembled from organic building blocks via strong covalent bonds can be divided into two subcategories: crystalline, including covalent organic frameworks (COFs), and amorphous, like conjugated microporous polymers (CMPs) and hypercrosslinked polymers (HCPs), porous organic cages (POCs), covalent triazine frameworks (CTFs), and porous aromatic frameworks (PAFs). With high stability, porosity, and designable structures, POFs have exhibited great potential for gas separation processes.^[3c]^

**Scheme 1:**
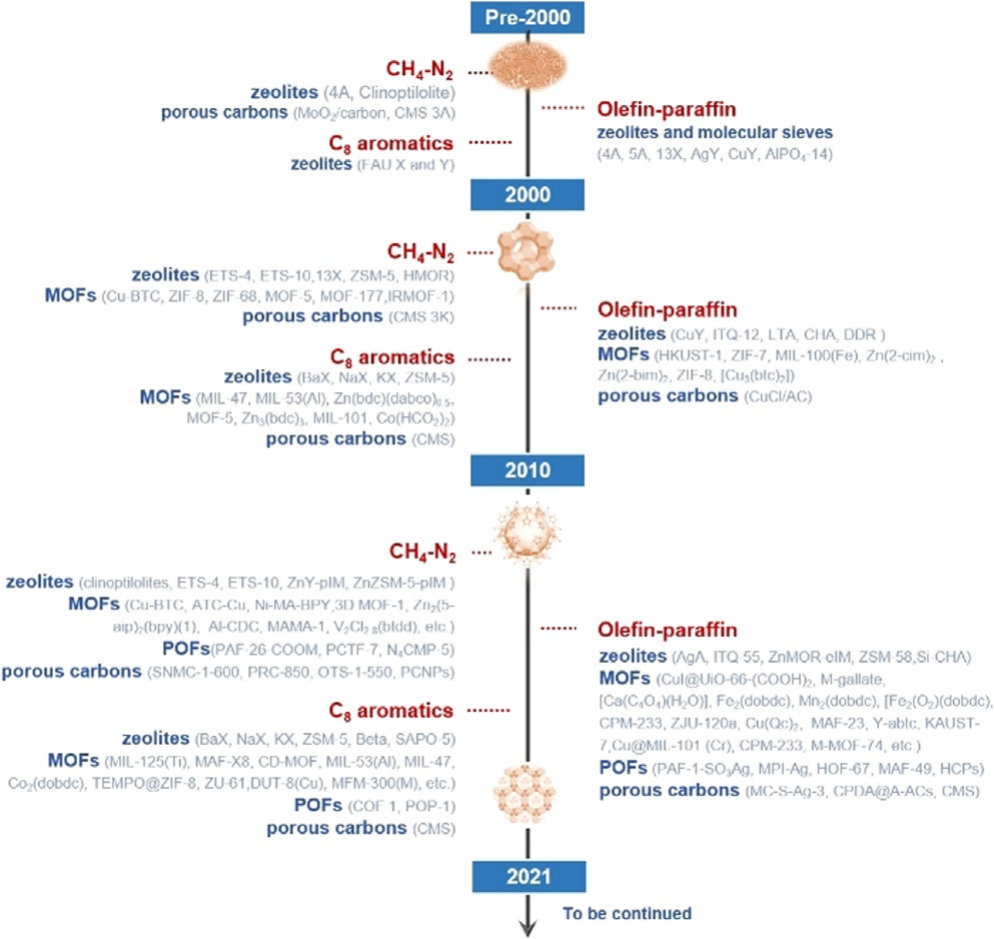
Timeline summarizing the trends in adsorptive hydrocarbon separation and purification with various porous materials over the latest three decades.

During the past three decades, a large amount of research has been devoted to the adsorptive separation and purification of hydrocarbons. Research on porous materials for hydrocarbon separation and purification has experienced an explosive growth (Scheme 1). In this Minireview, we summarize the latest trends and related developments of hydrocarbon purification and separation ranging from methane/nitrogen, ethene/ethane, propene/propane separation to the separation of C_8_ aromatics with porous adsorbents. This article is organized according to the different hydrocarbon separation systems with a further section focusing on the different porous materials and the different separation mechanisms. The future trends and challenges for each type of porous material are also discussed.

## 2 Mechanisms, Performance Evaluation, and Computational Methods

The adsorptive separation by the above‐mentioned porous materials is achieved by one of following three mechanisms: steric, kinetic, or equilibrium effects.^[20]^ Equilibrium separation processes are the most common, with a vast majority of adsorptive separation processes operating through the equilibrium adsorption of mixture components. Kinetic separation is achieved by virtue of the differences in diffusion rates of different molecules, of which air separation with carbon molecular sieves is a typical case. For steric effects, size or shape sieving can be achieved in porous materials with suitable pore size and geometries, in which small and properly shaped molecules can diffuse into the adsorbent, whereas molecules that are too large to enter the pores are totally excluded. The uniform channels and aperture size in the well‐defined crystalline structure offers zeolites and MOFs the possibility to separate different molecules by a size exclusive effect. Two typical cases of steric separation can be found in 3A zeolite for solvent drying and 5A zeolite for the separation of linear paraffins from branched‐chain and cyclic hydrocarbons.^[21]^ Benefiting from a variety of ordered pore structures and adjustable porosities, molecular sieving of ethylene over ethane,^[22]^ propene over propane,^[23]^ acetylene over ethylene,^[24]^ and methane and nitrogen from carbon dioxide^[25]^ have been successfully achieved by the finetuning of some MOF structures. Adsorption selectivity is one of the most significant parameters for the evaluation of an adsorbent. Ideal adsorbed solution theory (IAST), as developed by Myers and Prausnitz, is the most widely accepted theory to predict mixed‐gas adsorption isotherms, which are entirely based on single‐component adsorption isotherms.^[26]^ Accurately measured single‐component isotherms and an excellent fitting of adsorption isotherms for the measured data are required for the application of IAST to predict adsorption selectivity.^[27]^ Another method to determine the adsorption selectivity proposed by Knaebel takes the ratio of the Henry's law constants of the two components from the single‐component adsorption isotherms, which is also called Henry's law selectivity.^[27b]^ For an adsorbent based on kinetics, the kinetic selectivity is often calculated by the ratio of the diffusional time constants (*D*/*r* ^2^, calculated by the short‐time solution of the diffusion equation^[28]^) of the two gases. Breakthrough experiments are particularly useful in evaluating the practical separation performance of an adsorbent. In breakthrough experiments, a flowing gas with a well‐defined concentration of one or more adsorbates in a carrier gas passes through a fixed bed of porous adsorbents. A breakthrough curve is the time‐resolved effluent concentration of the adsorbate at the outlet of the fixed bed. The breakthrough results show clearly separation performance of the adsorbent.

Computational simulation has been widely applied in the study of adsorption and separation of porous adsorbents. One of the commonly used methods is quantum mechanics, such as ab initio and density functional theory (DFT). DFT has been a very popular method over the past years, in which the energy of a molecule can be determined in terms of the electron density instead of the electron wave function.^[29]^ Quantum mechanical methods can be used to determine the optimized position of an adsorbate molecule within a certain cluster extracted from the crystal structure of the porous material as well as the adsorption energy for an adsorbate molecule with the optimized binding site within the cluster with high accuracy.^[30]^ Another broadly used method is classical molecular simulations, such as molecular dynamics (MD) simulations and grand canonical Monte Carlo (GCMC) calculations. MD is a simulation of the time‐dependent behavior of a molecular system,^[31]^ which can be used to investigate the dynamic properties of an adsorbate in an adsorbent. GCMC is the most widely used molecular simulation method, which can be used to simulate gas adsorption in adsorbent over a wide range of temperatures and pressures.^[30]^ Adsorption isotherms, adsorption capacity, enthalpies of adsorption and selectivity (for mixtures) can be obtained from GCMC simulations.

Since there are thousands of porous materials (particularly MOFs), rapid screening of ideal and promising adsorbents can be difficult. High‐throughput computational screening (HTCS) has emerged as a powerful tool to make the fast evaluation and rational design of adsorbents feasible.^[32]^ One screening strategy is high‐throughput screening with molecular simulation or DFT calculations, which have played an important role in quickly identifying promising structures and accurately assessing adsorption and separation performances of porous adsorbents.^[32a]^ However, the accumulation of tremendous volumes of simulated data and the rapid growth of adsorbents (mainly MOFs) make this screening strategy inefficient and huge computational resources as well as valuable research time would be wasted.^[33]^ Another high‐throughput screening strategy based on machine learning that can overcome the above‐mentioned problems by the training of data has gradually received more and more attention.[^32b^, ^33^, ^34^]

## 3 Methane–Nitrogen Separation

CH_4_/N_2_ separation is intrinsically difficult because of the close kinetic diameters and comparable polarizability of CH_4_ and N_2_ (Table 1). For adsorption separation, according to the separation mechanisms, adsorbents can be divided into two categories: 1) CH_4_‐selective adsorbents, which exhibit stronger adsorption interactions and higher adsorption capacity for CH_4_ than N_2_; the separation is typically based on the equilibrium mechanism; 2) N_2_‐selective adsorbents, which preferentially adsorb N_2_ over CH_4_; the separation is dominantly based on the kinetic effect or steric effect. We will now discuss these two groups of materials separately.

**Table 1:** Physicochemical properties of a selection of molecules, including kinetic diameter, boiling point (B.p.), polarizability, dipole moment, and quadrupole moment.^[35]^

Gas molecule	Kinetic diameter [Å]	B.p. [K]	Polarizability [×10^−25^ cm^3^]	Dipole moment [D]	Quadrupole moment [×10^−26^ esu cm^2^]
CH_4_	3.8	109–113	26	0	0
N_2_	3.64	77.3	17.6	0	1.52
C_2_H_6_	4.44	184.5	44.7	0	0.65
C_2_H_4_	4.16	169.4	42.52	0	1.5
C_3_H_8_	5.3–5.12	231.1	62.9–63.7	0.083	0
C_3_H_6_	4.68	225.4	62.6	0.366	0
PX	6.7	411.5	137	0.1	–
MX	7.1	412.3	142	0.36	–
OX	7.4	417.6	149	0.649	–
EB	6.7	409.3	142	0.59	–

### 3.1 Methane‐Selective Adsorbents

Both CH_4_ and N_2_ are nonpolar molecules. For most porous materials, CH_4_ is always preferentially adsorbed over N_2_ due to its higher polarizability.^[36]^ Therefore, the mechanism of CH_4_‐selective adsorbents for CH_4_/N_2_ separation is predominantly equilibrium‐based. Porous carbons and MOFs are extensively studied porous materials for CH_4_/N_2_ separation.[^13b^, ^13e^, ^14c^, ^14d^, ^37^] Some zeolites have been tried and tested for CH_4_/N_2_ separation as well.^[38]^

#### 3.1.1 Carbonaceous Materials

Carbonaceous materials have been used for CH_4_/N_2_ separation for a long time. In earlier studies, porous carbons were treated by loading other chemicals, such as Br_2_, ICl, or MoO_2_, onto the adsorbents to improve their CH_4_/N_2_ separation performance.[^11a^, ^12a^] In recent years, heteroatom‐doped porous carbons and activated carbons derived from biomass have been explored.[^13b^, ^13e^, ^14c^, ^39^] N‐rich microporous carbons derived from N‐containing polymers were obtained by a solvent‐free method; they possessed narrow pore size distributions (ca. 0.5–3 nm) and achieved an IAST CH_4_/N_2_ selectivity of up to 5.1 at 298 K and 1 bar.^[14a]^ High N‐content porous carbons were also successfully synthesized from shrimp shells for CH_4_/N_2_ separation of coal‐bed gas.^[14b]^ The obtained activated carbons exhibited an IAST CH_4_/N_2_ selectivity of ≈5 at 298 K and 1 bar. Surface functionalization also has a strong effect on the separation performance of porous carbons. It was found that the activated carbons derived from bamboo sawdust had a higher adsorption capacity for CH_4_, while oxygen‐containing groups on activated carbons can improve the surface polarity and enhance the adsorption ability for N_2_, and thus the material has a lower IAST CH_4_/N_2_ selectivity.^[13c]^ Zhong et al. chose rice as a carbon source for making carbon‐based adsorbents and found that carboxyl groups are the dominant surface groups and responsible for the enhanced IAST CH_4_/N_2_ selectivity.^[13d]^ More recently, Lu et al. reported self‐pillared 2D polymer and ultramicroporous carbon plates prepared by a one‐pot multicomponent sequential assembly method.^[40]^ With the narrow ultramicropore size distribution (4.8 Å), the pillared polymer nanoplates exhibit a highly competitive CH_4_/N_2_ selectivity at lower CH_4_ partial pressure.

#### 3.1.2 Zeolites

**Figure 1:**
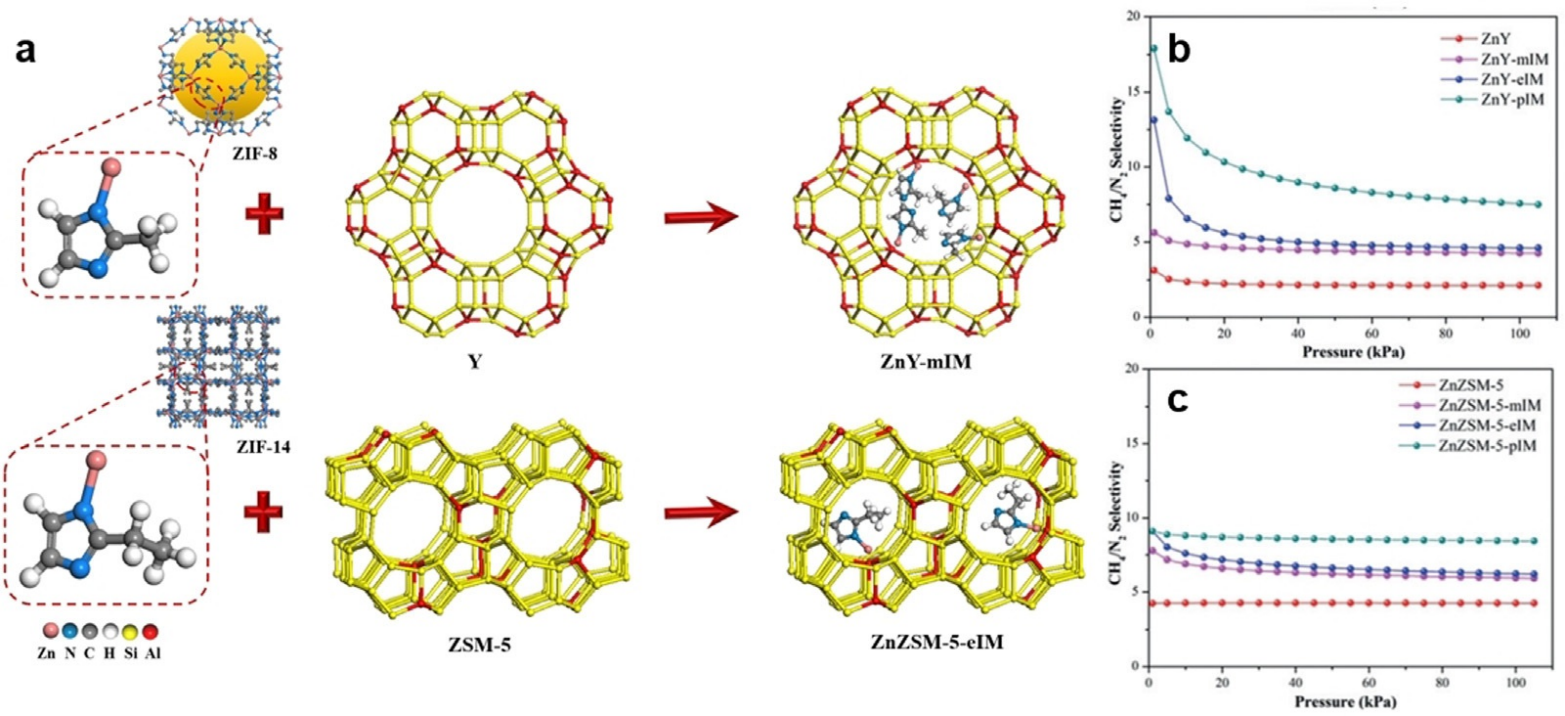
a) Schematic of zeolites decorated with ZIF‐8 and ZIF‐14 subunits. b) IAST selectivities of pristine and decorated zeolite Y and ZSM‐5 adsorbents for equimolar CH_4_/N_2_ mixtures at 298 K. Adapted from ref. [38e].

The separation performance of zeolites can be affected by the window size, pore geometry, and cation distribution of the zeolite material. Theoretically, by surface modification and pore size adjustment of zeolites, gas mixtures with similar compositions can be separated. However, precise adjustment is rather difficult to achieve. The earliest application of zeolites used for CH_4_/N_2_ separation can be traced back to 1958.^[41]^ Some traditional zeolites, including zeolite 4A,^[42]^ zeolite 5A,^[43]^ ZSM‐5,[^38c^, ^44^] 13X,^[45]^ HMOR, and chabazite,^[42a]^ were studied and tested for the separation of CH_4_/N_2_. The separation results indicated that their separation performances towards CH_4_/N_2_ were rather desirable. Consequently, traditional zeolites are not applicable in CH_4_/N_2_ separation. On the other hand, modification of traditional zeolites can be an effective method to improve their CH_4_/N_2_ separation performances. Liu et al. report a strategy of introducing subunits of ZIFs into zeolites Y and ZSM‐5 to obtain effective adsorbents with advantages of both zeolites and MOFs (Figure 1).^[38e]^ Simulation results suggested that the incorporation of ZIF subunits (Zn‐mIM, Zn‐eIM, and Zn‐pIM) may result in higher CH_4_/N_2_ selectivities. Experimental results validated that the incorporation of ZIF subunits into the zeolite structure lead to an increase in the IAST CH_4_/N_2_ selectivity, which reached a value of 8.4.

#### 3.1.3 Metal–Organic Frameworks

**Figure 2:**
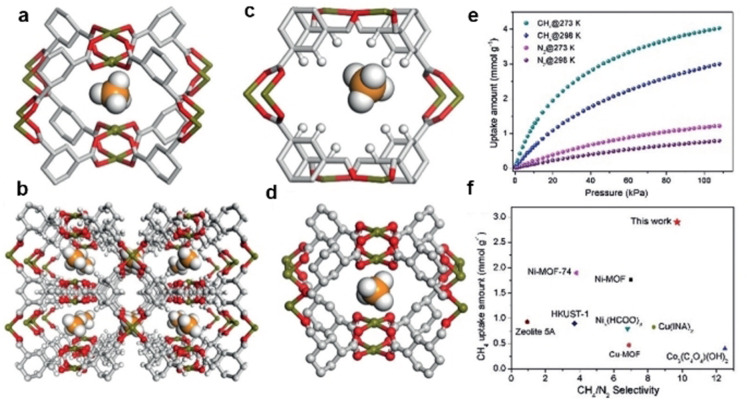
a) Binding site I for methane between adjacent Cu‐metal sites and c) Binding site II for methane in the aliphatic hydrocarbon cavity, as determined by modeling studies conducted on the ATC‐Cu material. b) Single‐crystal structure of methane loaded in ATC‐Cu viewed along the *a* axis and d) close‐up view. e) Methane and nitrogen adsorption isotherms for ATC‐Cu at 273 and 298 K. f) CH_4_/N_2_ selectivity for high‐performance materials at 1 bar and 298 K. Adapted from ref. [47].

Since the 20^th^ century, MOFs have been tested in great detail for their potential in CH_4_/N_2_ separation^[46]^ and progress has been made in recent years. Various methods, including synthesizing MOFs with new framework structures, functionalizing MOFs with different groups, and reacting and combining MOFs with other chemicals or materials, have been investigated for the improvement of CH_4_/N_2_ separation performance; these methods primarily aim at increasing the interaction between CH_4_ and the adsorbent. A MOF‐based methane nanotrap (ATC‐Cu) was reported featuring oppositely adjacent open metal sites and dense alkyl groups that can induce strong interactions with methane (Figure 2).^[47]^ Single‐crystal X‐ray diffraction experiments and molecular simulation studies indicated that ATC‐Cu provides very strong binding sites for methane, attributing to a IAST CH_4_/N_2_ selectivity up to 9.7 at 298 K and 1 bar. Woo et al. constructed a new MOF with a 3D framework with alternating large and small channels along the a and b directions, which has an IAST CH_4_/N_2_ selectivity of 7.0 at 298 K and 1 bar.^[37a]^

**Figure 3:**
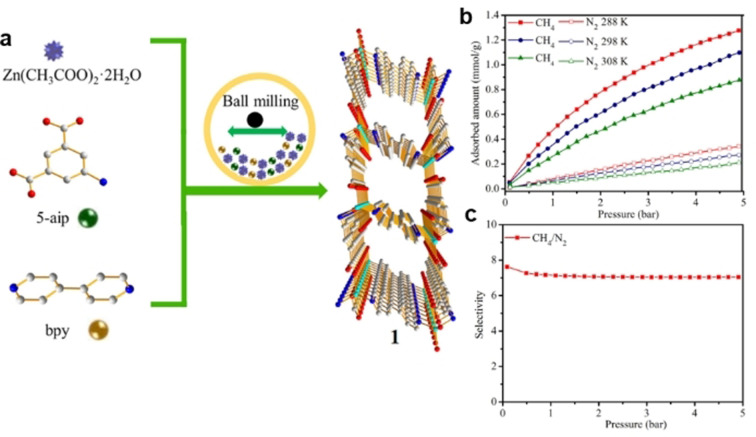
a) Schematic of the mechanochemical method for the rapid synthesis of Zn_2_(5‐aip)_2_(bpy). b) CH_4_ and N_2_ adsorption isotherms of Zn_2_(5‐aip)_2_(bpy) at 288, 298, and 308 K. c) IAST selectivity of Zn_2_(5‐aip)_2_(bpy) measured with ah equimolar CH_4_/N_2_ mixture at 298 K. Adapted from ref. [37c].

Several MOFs with layered and pillared structures proved to be efficient CH_4_/N_2_ adsorbents due to their narrow and uniform pore networks. Two isostructural MOF materials, Co‐MA‐BPY and Ni‐MA‐BPY, with intriguing pillar‐layer structures, have prominent IAST CH_4_/N_2_ selectivities of 7.2 and 7.4 (CH_4_/N_2_=50/50,v/v), respectively, at 298 K and 1 bar.^[48]^ Lately, the zinc‐based pillar‐layer MOF Zn_2_(5‐aip)_2_(bpy) also has been effectively used for CH_4_/N_2_ separation (Figure 3).^[37c]^ Molecular simulation indicated that within its narrow pore environment, the spheroidal molecular structure of CH_4_ could be more adequately packed than the linear molecular structure of N_2_. As a result, a sample achieved IAST CH_4_/N_2_ selectivity of up to 7.1 at 298 K and 1 bar. Based on the saturated C‐H bonds as well as the corresponding *trans* corner in the ligand, Liu et al. constructed MOFs with specific cages to preferentially adsorb CH_4_ molecules. In an Al‐CDC MOF, the aliphatic ligand with low polarity that contains saturated C‐H bonds may have a relatively strong interaction with CH_4_, leading to a high IAST CH_4_/N_2_ selectivity of 13.1 at 298 K and 1 bar.^[49]^

Modification of MOFs via compositing MOFs with other chemicals or materials is likewise an effective way to elevate their CH_4_/N_2_ separation performance. Doping Mg^2+^ into MIL‐101 has been investigated to enhance the selective adsorption of CH_4_/N_2_ of MIL‐101.^[37d]^ Doping Mg^2+^ increases the adsorption capacity of CH_4_ and N_2_ at different levels because doping the proper amount of Mg^2+^ restrains the generation of H bonds, which has a positive effect on methane gas adsorption. The IAST CH_4_/N_2_ selectivities upon doping MIL‐101 with Mg^2+^ also greatly increased, from ≈2.1 for pristine MIL‐101 to ≈4.5 for MIL‐101@12.8 % Mg^2+^ (CH_4_/N_2_=0.3/0.7, 298 K, 60 bar). Uzun et al. incorporated ionic liquids (ILs), 1‐*N*‐butyl‐3‐methylimidazolium hexafluorophosphate, [BMIM][PF_6_], and its methylated form, [BMMIM][PF_6_], in CuBTC to examine the effect of methylation of ILs on the gas separation performance.^[50]^ Compared to the corresponding selectivities of pristine CuBTC, CH_4_/N_2_ selectivities of the two composites, [BMIM][PF_6_]/CuBTC and [BMMIM][PF_6_]/CuBTC, increased by 50 % and 60 %, respectively, at 298 K and 1 bar.

#### 3.1.4 Porous Organic Frameworks

Porous organic frameworks with higher chemical stability and specific surface areas have also attracted attention in CH_4_/N_2_ separation.^[51]^ Rational modification with variable functionalities has been used to modify the physicochemical properties of POFs, aiming at improving their gas adsorption capacity and selectivity. Upon introduction of light metal ions to the porous aromatic framework (PAF), the adsorption affinity of PAF‐26‐COOM to CH_4_ gases is enhanced compared to pristine PAF‐26‐COOH.^[51a]^ The improved performance of PAF‐26‐COOM and significantly higher CH_4_/N_2_ selectivity is attributed to the strong interaction between CH_4_ molecules and PAF‐26‐COOM. A series of adamantane porous covalent triazine‐based frameworks (PCTFs) with varying symmetry and functional group density were prepared by using a Lewis acid catalyst and a strong Brønsted acid catalyst, respectively.^[51b]^ The most selective PCTF‐7 is found to have a high CH_4_/N_2_ selectivity of 7 at 273 K and 1 bar. Table 2 provides an overview of selected state‐of‐the‐art porous materials for CH_4_/N_2_ separation.

**Table 2:** Overview of selected state‐of‐the‐art porous materials for CH_4_/N_2_ separation. Sel.: selectivity.

Material	Sel.	*T* [K]	*P* [bar]	CH_4_ uptake [mmol g^−1^]	Ref.
SNMC‐1‐600	5.1	298	1	1.45	[14a]
SA‐1‐700	5	298	1	1.53	[14b]
AC‐1.5‐400	5.2	298	1	0.6	[13c]
PRC‐850	5.7	298	1	1.12	[14d]
NAPC‐1‐6	5.5	298	1	1.0	[13b]
OTS‐1‐550	5.9	298	1	1.12	[13e]
OTSS‐2‐450	4.9	298	1	0.93	[14c]
PCNPs	10	298	1	1.17	[40]
Linde 4A	2.4	273	1	0.97	[42a]
H^+^ mordenite	2.8	273	1	0.92	[42a]
chabazite	1.5	273	1	1.31	[42a]
5A	0.94	298	1	≈0.95	[43a]
H‐ZSM‐5‐30	2.97	313	1	0.71	[38c]
ZnZSM‐5‐pIM	8.4	298	1	0.25	[38e]
ATC‐Cu	9.7	298	1	2.9	[47]
3D MOF‐1	7.0	298	1	1.15	[37a]
Ni‐MA‐BPY	7.4	298	1	1.01	[48]
Zn_2_(5‐aip)_2_(bpy) (1)	7.1	298	1	≈0.35	[37c]
Al‐CDC	13.1	298	1	1.43	[49]
[BMMIM][PF_6_]/CuBTC	5.2	298	1	≈0.74	[50]
PAF‐26‐COOMg	6.5	298	1	–	[51a]
PCTF‐7	6	273	1	0.65	[51b]
N_4_CMP‐5	10.5	273	1	≈0.5	[51c]
boron nitride	4.6	298	1	0.65	[52]
Ni‐MOF‐4	1.35	298	1	2.54	[53]

### 3.2 Nitrogen‐Selective Adsorbents

While the majority of adsorbents for CH_4_/N_2_ separation are equilibrium‐based, a proportion of materials separate CH_4_/N_2_ mixtures based on kinetic variance. Due to the difference in the kinetic diameters of CH_4_ and N_2_, it should be possible to separate them based on a kinetic mechanism. Therefore, many studies on CH_4_/N_2_ separation also focused on sorbents for kinetic separation (e.g., carbon molecular sieves, clinoptilolites and some MOFs).

Caron molecular sieves (CMS) is a type of carbonaceous material, whose molecular sieving properties depends on their narrow and uniform pore size distribution. Based on their special pore textures, CMS have been successfully used for separating N_2_ from CH_4_/N_2_ mixtures.[^11b^, ^54^] As early as 1991, the diffusion time constants of N_2_ and CH_4_ in CMS 3A were reported by Ma et al.; the adsorbent was found to exhibit selectivity for N_2_/CH_4_ separation.^[55]^ In 2016, Li et al. used CMS to concentrate methane from raw gas of 10 % CH_4_ to 79 % purity during a high‐pressure adsorption step with 93 % recovery.^[54b]^ Recent research also found that CMS materials designed with proper microporosity would benefit practical coal mine methane upgrading.^[54c]^ Clinoptilolites and titanosilicates are two promising adsorbents for the kinetic separation of N_2_/CH_4_ mixtures. Clinoptilolites are naturally occurring zeolites with a 2D channel structure formed by eight‐membered rings and ten‐membered rings. The location, number, and type of cations in these channels have a heavy impact on the selectivity and adsorption rate of gases.^[56]^ The separation performances of clinoptilolites modified by cation exchange are different depending on the metal cations. Among the many metal cations, Na^+^, Mg^2+^, Li^+^, and Ni^2+^ clinoptilolites showed favorable kinetic selectivity for possible N_2_/CH_4_ kinetic separation. Modified titanosilicate molecular sieves, including ETS‐4, ETS‐10, and UPRM‐5, are another representative class of adsorbents with desirable N_2_/CH_4_ kinetic selectivities.^[57]^

**Figure 4:**
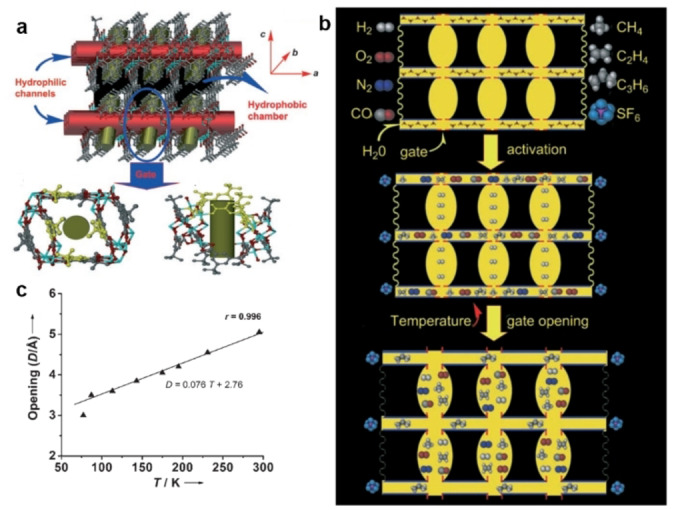
a) Structure of MAMS‐1. b) Schematic of the gating effect mechanism in MAMS‐1. c) Temperature‐dependent gate opening of MAMS‐1. ▴: experimental values; —: linear fit. Adapted from ref. [59].

MOFs with flexible framework structures have attracted enormous attention and some flexible MOFs preferentially adsorb N_2_ over CH_4_. Zhou et al. synthesized a mesh‐adjustable MAMS‐1 from H_2_(bbdc) and Ni(NO_3_)_2_ for gas separations. As shown in Figure 4, MAMS‐1 has a flexible structure and its gates open linearly as temperature increases.^[58]^ The adjustable mesh of MAMS‐1 makes it possible to separate gases with kinetic diameters in the range of 2.9 to 5.0 Å. At 113 K, the gate of MAMS‐1 opens to about 3.7 Å, which is wide enough to allow N_2_ (3.64 Å) to enter the chambers, but CH_4_ (3.8 Å) stays in the hydrophilic channels.

**Figure 5:**
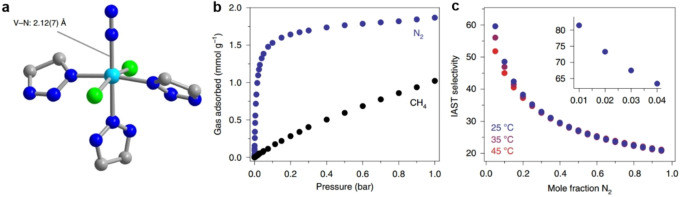
a) Structure of a single vanadium site in V_2_Cl_2.8_(btdd) after dosing with 700 mbar of N_2_, as determined from analysis of powder X‐ray diffraction data. Cyan, green, blue and gray spheres represent V, Cl, N and C atoms, respectively; a 40 %‐occupied terminal chloride ligand has been omitted. b) Adsorption isotherms for N_2_ (blue) and CH_4_ (black) collected at 25 °C in V_2_Cl_2.8_(btdd). c) IAST selectivity values calculated at 25, 35, and 45 °C for various N_2_:CH_4_ ratios at a total pressure of 1 bar. Adapted from ref. [61].

Featuring low‐energy, π‐symmetric orbitals capable of accepting electron density, N_2_ is a weakly π‐acidic species. Based on quantum mechanical computations, the team of Jeffrey R. Long predicted that V‐MOF‐74 can be used to separate dinitrogen from methane due to the selective π back‐bonding interactions between the vanadium(II) cation centers in this MOF and the unoccupied π* orbitals of N_2_.^[59]^ This insight provides new MOF targets to synthesize. In 2013, inspired by biomimetic nitrogen fixation to produce ammonia, they found that mesoporous MOF containing accessible Cr^III^ sites is able to thermodynamically capture N_2_ selectively over CH_4_ and O_2_; the presence of unsaturated Cr^III^ sites allows a much stronger binding of N_2_ over the two other gases.^[60]^ Recently, Long and his group reported the synthesis of a metal–organic framework with exposed vanadium(II) sites, which engages π‐acidic gases via backbonding interactions.^[61]^ The N_2_/CH_4_ separation performance of V^II^‐MOF has been verified. Specifically, the btdd^2−^ ligand was used to synthesis V_2_Cl_2.8_(btdd) instead of more common carboxylate‐containing ligands to achieve better square‐pyramidal vanadium(II) centers. The IAST N_2_/CH_4_ selectivity values of V_2_Cl_2.8_(btdd) are exceptional for low N_2_ concentrations at 1 bar total pressure (Figure 5 c). The N_2_/CH_4_ selectivity is 38 for a 20:80 N_2_/CH_4_ mixture at 25 °C and at 2:98 N_2_/CH_4_, the selectivity reaches 72. Incorporating such π‐basic metal centers into porous materials offers a handle for capturing and activating key molecular species within next‐generation adsorbents.

## 4 Olefin–Paraffin Separation

Ethene/ethane and propene/propane separations are the most important separation processes among olefin/paraffin mixtures as ethene and propene are the most important raw materials in the petrochemical industry. The very similar boiling point and the small variations in the condensabilities of these molecules (ethene/ethane and propene/propane) lead to great challenges in those separations, and also makes the currently used cryogenic distillation based on different vapor pressures and boiling points very energy‐intensive. Consequently, adsorption‐based separation processes are explored and developed.

### 4.1 Ethene/Ethane Separation

#### 4.1.1 Ethene‐Selective Adsorbents

With the presence of π electrons, higher quadrupole moment, and smaller molecular size, ethene is more easily adsorbed and also adsorbed in higher amounts than ethane by most of the developed adsorbents.^[62]^ In this regard, a large number of porous adsorbents exhibit excellent performance for ethene/ethane separation based on three possible mechanisms: equilibrium‐based, kinetic‐based, and size exclusion mechanisms.

**Figure 6:**
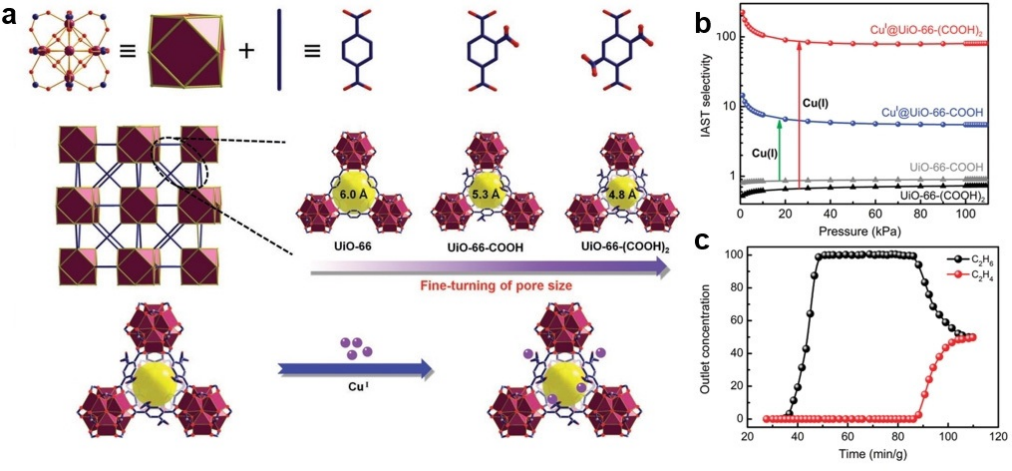
a) X‐ray single‐crystal structure of UiO‐66‐type MOFs. The pore window size can be systemically modulated by the judicious choice of organic linkers and it can be further contracted after the configuration of copper(I) ions. b) IAST calculations of activated UiO‐66‐type MOFs for C_2_H_4_/C_2_H_6_ separation at 298 K. c) Breakthrough curves of CuΙ@UiO‐66‐(COOH)_2_ for C_2_H_4_/C_2_H_6_ (50/50, v/v) separation at 298 K. Adapted from ref. [65a].

**Equilibrium‐based mechanism**. Ethene/ethane separation achieved by thermodynamically driven separation is one of the most common and popular cases. Owing to the π‐complexation effect between unsaturated hydrocarbons and metal ions (mostly Cu^I^ and Ag^I^), ethene undergoes selective π‐complexation with metal ions in adsorbents (porous carbons, zeolites, MOFs, and POFs) or the open metal sites (OMSs) in MOFs and is thus separated from ethane‐containing mixtures. One effective method, the incorporation of metal ions into the pores of adsorbents, has been implemented in and well proven for different types of porous materials, including zeolites, porous carbons, and MOFs. Zeolites, in particular cation‐exchanged zeolites, have been successfully used for ethene/ethane separation.^[63]^ Porous carbons and mesoporous silica were also used as supports for Cu^I^ and Ag^I^ loading to separate ethene/ethane.[^12b^, ^64^] MOFs and POFs, in which Cu^I^ is chelated by organic linkers (Figure 6) and that are functionalized with Ag^I^ by grafting or sulfonate functionalization, were shown to have superior selectivity towards ethene.[^19e^, ^65^] Another approach is to achieve π‐complexation between open metal sites in MOFs and ethene. MOFs can contain coordinatively unsaturated sites or open metal sites when vacant Lewis acid sites on the metal ions or cluster nodes have been generated.^[66]^ Due to the π‐interactions between the electron‐rich π‐orbital in olefins and the vacant s‐orbital of the open metal site, MOFs with open metal sites preferentially adsorb olefins over paraffin, achieving outstanding olefin/paraffin separation performance. The open metal sites in coordinatively unsaturated MOFs have selective interactions with olefins via π‐complexation.^[67]^ CuBTC is the first MOF with open metal sites for the efficient separation of ethene/ethane.^[68]^ M_2_(dobdc) frameworks (also MOF‐74, M: Mn, Fe, Co, Ni, Zn; dobdc^4−^: 2,5‐dioxido‐1,4‐benzenedicarboxylate) with a high density of open metal sites were also explored and exhibited great potential in ethene/ethane separation.^[69]^ The strong π‐interaction leads to high gas uptake and separation selectivity, but the regeneration of these adsorbents can also be difficult and highly energy consuming.

**Figure 7:**
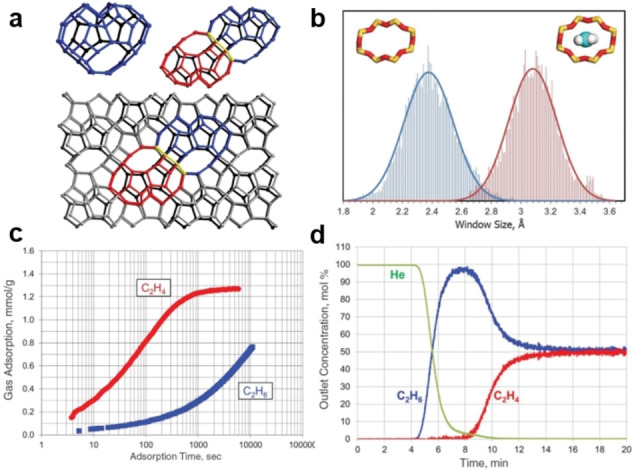
a) Zeolitic structure and b) window size of ITQ‐55. c) Time‐dependent C_2_H_4_ and C_2_H_6_ uptake profiles of ITQ‐55, at 30 °C and 0.45 bar and 0.6 bar, respectively. d) Breakthrough curves of ITQ‐55 for C_2_H_4_/C_2_H_6_ (50:50) at 50 °C and 8.5 bar. Adapted from ref. [70].

**Kinetic‐based mechanism**. Based on the difference in the shape and size of ethene and ethane molecules, a number of adsorbents have been developed for ethene/ethane separation. For adsorbents based on kinetics, pore dimensions and pore shape play dominant role in the overall separation performance. Corma et al. synthesize a pure silica zeolite with large heart‐shaped cages and framework flexibility, which can kinetically separate ethene from ethane with an exceptional selectivity of ≈100 (Figure 7).^[70]^ When ethene molecules enter the center of the 8‐ring window of ITQ‐55, the window size will expand from 2.38 Å of the empty structure to 3.08 Å (Figure 7 b). However, the adsorption capacity is also limited by its contracted aperture. A robust MOF GT‐18 with optimum pore size and shape was synthesized through a mixed‐linker strategy and displayed promising diffusion selectivity toward ethylene.^[71]^ With a benzotriazole (BTA)/benzimidazole (BIM) linker synthesis ratio of 4:1, GT‐18 was obtained, featuring a 10‐ring window and flexible pore apertures (≈3 Å). The phosphate‐anion pillared MOF ZnAtzPO_4_ decorated with electronegative groups was also reported; its periodically expanded and contracted aperture enables effective trapping of C_2_H_4_ and impedes the diffusion of C_2_H_6_. An equilibrium–kinetics synergetic effect was observed in this MOF, which displayed a combined selectivity of 32.4 at 273 K and 1 bar.^[72]^

**Figure 8:**
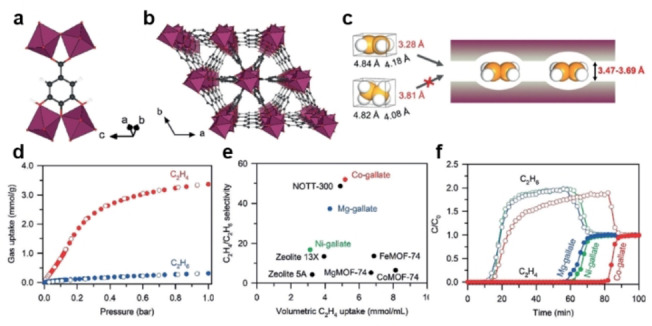
a) Coordination environment of the gallate ligand and MO_6_. b) Perspective view of the structure along the *c* axis showing the triangular main channels and the regular branched channels leaning against the main ones. c) Diagram of the fusiform branched channels. d) Single‐component adsorption isotherms of Co‐gallate at 298 K. e) Comparison of C_2_H_4_/C_2_H_6_ adsorption selectivity and volumetric C_2_H_4_ uptake at 1 bar in M‐gallate and other best‐performing materials. f) Breakthrough curves of M‐gallate for the equimolar C_2_H_4_/C_2_H_6_ mixture at 273 K and 1 bar. Adapted from ref. [73].

**Size exclusion mechanism**. Size exclusion or molecular sieving is an ideal approach for separation, by which only small and properly shaped molecules can diffuse into the adsorbent, allowing a highly selective separation based on molecular size or shape cut‐off. Studies aimed at molecular sieving mainly focus on the difference between the kinetic diameters of adsorbate molecules, the pore size of the adsorbent, and differentiation of van der Waals molecular dimensions and molecular cross‐section. Focusing on the difference in molecular size and shape of ethene and ethane, Chen et al. reported an ultramicroporous MOF [Ca(C_4_O_4_)(H_2_O)] with aperture sizes (with slightly different shapes of 3.2×4.5 Å^2^ and 3.8×3.8 Å^2^) similar to the size of ethene molecules but smaller than the ethane molecules.^[22]^ Owing to the good size/shape match and its highly rigid pore structure, [Ca(C_4_O_4_)(H_2_O)] can act as a molecular sieve to prevent the transport of ethane inside its channels. The low cost of the raw materials (calcium nitrate and squaric acid) and the high stability of [Ca(C_4_O_4_)(H_2_O)] make it promising for industrial applications. However, whether the coordinated water molecules will be lost after long‐term use and the effect of this on the structural stability remains to be considered. Considering the differentiation of molecular cross‐section size, a family of gallate‐based MOFs, M‐gallate (M=Ni, Mg, Co) featuring 3D interconnected zigzag channels and aperture sizes of 3.47–3.69 Å, also exhibited ideal exclusion of ethene and ethane (Figure 8).^[73]^ The special channels and pores can ideally separate ethene (3.28×4.18×4.84 Å) and ethane (3.81×4.08×4.82 Å) through molecular cross‐section size differentiation (Figure 8 c). Consequently, for Co‐gallate, an unprecedented IAST ethene/ethane selectivity of 52 was achieved at 298 K and 1 bar for equimolar ethene/ethane mixtures. Table 3 provides an overview of selected state‐of‐the‐art porous materials for C_2_H_4_/C_2_H_6_ separation.

**Table 3:** Overview of selected state‐of‐the‐art porous materials for C_2_H_4_/C_2_H_6_ separation. Sel.: selectivity.

Material	Sel.	*T* [K]	*P* [bar]	C_2_H_4_ uptake [mmol g^−1^]	Ref.
AgA	absolute	303	1	2	[63]
CuCl(8.0)/AC	69.42	303	1	2.57	[12b]
Ag‐Amberlyst 35	60.8	303	1	0.59	[64b]
PAF‐1‐SO_3_Ag	27	296	1	4.1	[19e]
MPI‐Ag	3.1	298	1	0.82	[65d]
CuI@UiO‐66‐(COOH)_2_	80.8	298	1	1.86	[65a]
CuBTC	2	298	1	≈7	[68]
Fe_2_(dobdc)	13	318	1	6.02	[69b]
Mn_2_(dobdc)	≈7	318	1	≈6.0	[69c]
Fe_2_(m‐dobdc)	25	298	1	≈7.2	[69d]

#### 4.1.2 Ethane‐Selective Adsorbents

Ethene/ethane separation by ethene‐selective adsorbents to obtain high‐purity ethene requires a two‐step “adsorption–desorption” process and subsequent multiple adsorption–desorption purification cycles, which are highly energy‐consuming. Therefore, ethane‐selective adsorbents would be more desirable and efficient for ethene/ethane separation, because ethene would be obtained directly which would thus be more energy‐saving. However, the development of ethane‐selective adsorbents is a challenging task. Ethene shows a larger quadrupole moment than ethane (ethene: 1.50×10^−26^ esu cm^2^, ethane: 0.65×10^−26^ esu cm^2^), while ethane has a higher polarizability (ethene: 42.52×10^−25^ cm^3^, ethane: 44.7×10^−25^ cm^3^) (Table 1). Therefore, unlike ethene‐selective adsorbents with strong adsorption sites for ethene, ethane‐selective adsorbents always suffer from poor selectivity due to the lack of strong adsorption sites. In spite the challenges, research on ethane‐selective adsorbents, predominantly MOF and POF materials, has been made significant progress over the several past years.^[74]^

**Figure 9:**
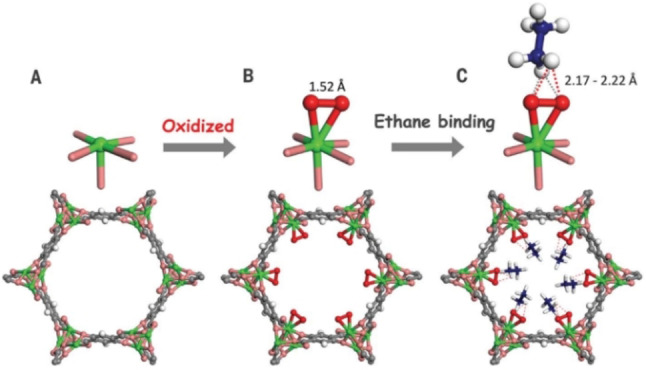
Structures of A) Fe_2_(dobdc), B) Fe_2_(O_2_)(dobdc), and C) Fe_2_(O_2_)(dobdc)⊃C_2_D_6_ at 7 K. Note the change from the open Fe^II^ site to the Fe^III^‐peroxo site for the preferential binding of ethane. Fe, green; C, dark gray; O, pink; O_2_^2−^, red; H or D, white; C in C_2_D_6_, blue. Adapted from ref. [74c].

Strengthening binding affinity towards ethane, enhancing host–guest (ethane) interactions, and decreasing or preventing strong adsorption sites for ethene are the most used and effective strategies to improve the separation performance of ethane‐selective adsorbents. The proper positioning of multiple electronegative and electropositive functional groups on the pore surface of the material MAF‐49 resulted in multiple and stronger C−H⋅⋅⋅N hydrogen bonds between ethane molecules and the MAF‐49 framework, leading to the preferential adsorption of ethane over ethene and a high IAST ethane/ethene selectivity of 9 for equimolar ethane/ethene mixtures with a relatively low ethane uptake (1.7 mmol g^−1^) at 316 K and 1 bar.^[74a]^ By controlling pore structures, the material Cu(Qc)_2_ (Qc^−^=quinolone‐5‐carboxylate) with a weakly polar pore surface exhibited self‐adaptive sorption behavior for ethane and thus higher binding affinity towards ethane over ethene.^[74b]^ At 298 K and 1 bar, it presented a IAST selectivity of 3.4 for equimolar ethane/ethene mixtures. Chen et al. reported the microporous MOF Fe_2_(O_2_)(dobdc) (dobdc^4−^: 2,5‐dioxido‐1,4‐benzenedicarboxylate), which displayed highly selective separation of ethane/ethene.^[74c]^ The Fe‐peroxo sites on the pore surface of Fe_2_(O_2_)(dobdc) a play key role in the recognition of ethane, which resulted in the adsorption of a larger amount of ethane than of ethene (Figure 9). At 298 K and 1 bar, the ethane uptake and the selectivity for an equimolar ethane/ethene mixture were 3.32 mmol g^−1^ and 4.4, respectively.

**Figure 10:**
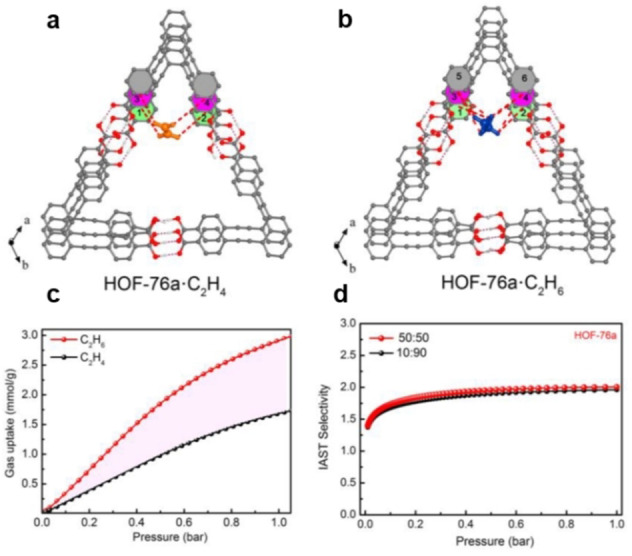
Comparison of the preferential adsorption sites for ethene (a) and ethane (b), and the close van der Waals contacts within the corner surface of triangular channel‐like pores, as obtained by DFT calculations (C, dark gray; O, red; H, white); C−H⋅⋅⋅π interactions highlighted by red dashed bonds, c) Adsorption isotherms of C_2_H_6_ (red) and C_2_H_4_ (black) for HOF‐76a at 296 K. d) IAST selectivity of HOF‐76a from C_2_H_6_/C_2_H_4_ (50/50 and 10/90) gas mixtures at 296 K. Adapted from ref. [62].

The novel hydrogen‐bonded organic framework (HOF) HOF‐76a has been exploited for ethane/ethene separation (Figure 10).^[62]^ The nonpolar/inert pore surface together with the triangular channel‐like pores within HOF‐76a enables it to preferentially adsorb ethane over ethene. The IAST selectivity for an equimolar ethane/ethene mixture is 2, at 296 K and 1 bar. The ethane‐selective MOF JNU‐2 with cage‐like cavities interconnected by a small aperture (≈3.7 Å) has been reported by Li et al.^[74d]^ The multiple C−H⋅⋅⋅O hydrogen bonds between ethane and the precise arrangement of oxygen atoms on the small aperture resulted in an enhanced ethane‐selectivity over ethene. Via a pore‐space‐partition (PSP) strategy, a family of heterometallic vanadium and titanium MOFs were synthesized.^[74e]^ The total annihilation of open metal sites in these MOFs could be beneficial for the ethane‐selective separation. Even though the lower adsorption enthalpy (21.9–30.4 kJ mol^−1^) is lower than that of MAF‐49 (−60 kJ mol^−1^)^[74a]^ and Fe_2_(O_2_)(dobdc) (−67 kJ mol^−1^),^[74c]^ six of these MOFs displayed remarkable ethane uptakes from 6.88 to 7.45 mmol g^−1^ at 298 K and 1 bar, while their ethane/ethene selectivities are moderate (≈1.75).

**Figure 11:**
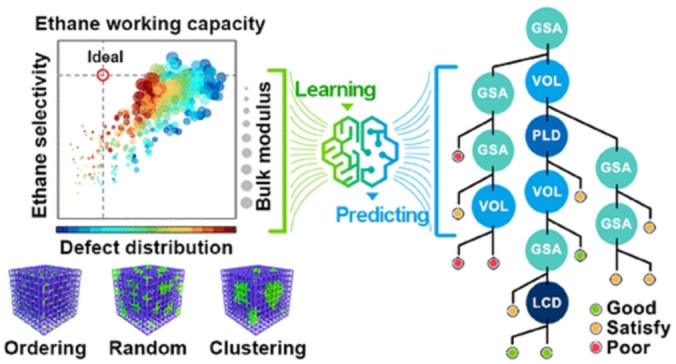
Scheme describing how machine learning is used to obtain insights into UiO‐66 with defects for enhanced ethane/ethene separation. Adapted from ref. [77a].

It is worth mentioning that high‐throughput computational screening (HTCS) methods have become a useful approach to screen adsorbents for gas adsorption and separations. In 2016, molecular simulation based computational screening of 278 different MOFs was performed to simulate their separation of ethane/ethene.^[75]^ Several MOFs were predicted to exhibit higher adsorption capacities and selectivities than zeolites under similar conditions. With the continuous increase in the number of adsorbents, larger databases have been screened. A large set of MOFs was computationally screened by first excluding MOFs with disordered atoms, open metal sites, and a pore limiting diameter <3.8 Å to identify ideal adsorbents for ethane/ethene separation.^[76]^ 16 ideal MOFs with ethane/ethene selectivity ≥2.16 and ethane uptakes ≥0.54 mmol g^−1^ were identified. In addition to molecular simulation based HTCS, a more effective machine learning based HTCS is beginning to emerge for hydrocarbon separations.^[77]^ Xi et al. created a modeling library of 425 UiO‐66 materials with a large variety of missing‐linker defects to explore the effect of defects in MOFs.^[77a]^ They proved that machine learning could be an efficient way to elucidate how the defects control the performance of UiO‐66 in adsorption, separation, and mechanical stability. This study also concluded that the concentration of defects is more important than their distribution in the overall separation performance of UiO‐66 materials. Finally, they provided a straightforward guide to access a privileged defect‐containing UiO‐66 material with optimal properties (Figure 11). By combining machine learning algorithms with molecular simulation, they screened the hypothetical metal–organic framework (h‐MOF) database for ethane/ethene separation materials.^[77b]^ Finally, four h‐MOF materials with the best ethane/ethene separation performance were identified by using the random forest (RF) algorithm. With the increasing number of possible porous materials, HTCS has been regarded as a valuable tool to identify the top‐performing materials from the large database of candidates and will accelerate the rational design and development of highly efficient adsorbents. Table 4 provides an overview of selected state‐of‐the‐art porous materials for C_2_H_6_/C_2_H_4_ separation.

**Table 4:** Overview of selected state‐of‐the‐art porous materials for C_2_H_6_/C_2_H_4_ separation. Sel.: selectivity.

Material	Sel.	*T* [K]	*P* [bar]	C_2_H_6_ uptake [mmol g^−1^]	Ref.
HOF‐76a	2	296	1	2.95	[62]
MAF‐49	2.73	298	1	1.73	[74a]
Cu(Qc)_2_	3.4	298	1	1.85	[74b]
Fe_2_(O_2_)(dobdc)	4.4	298	1	3.32	[74c]
CPM‐233	1.64	298	1	7.45	[74e]
CPM‐733	1.75	298	1	7.13	[74e]
ZJU‐120a	2.74	296	1	4.91	[74f]
MUF‐15	1.96	293	1	4.69	[74i]
PCN‐250	1.9	298	1	5.21	[74j]
ZIF‐7	1.5	298	1	1.83	[74k]
IRMOF‐8	1.8	298	1	4.3	[74l]

### 4.2 Propene/Propane Separation

**Figure 12:**
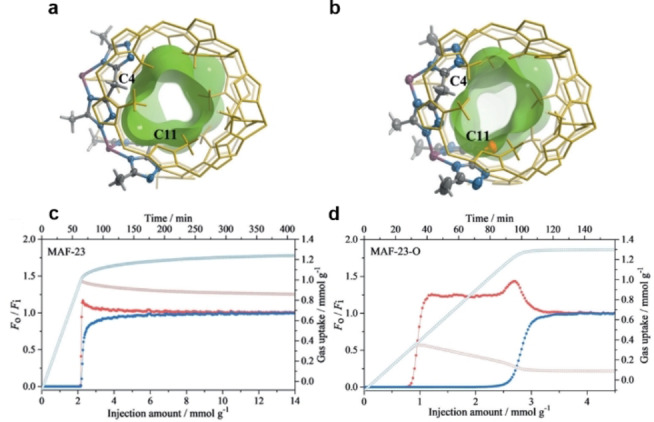
Crystal and pore structures of a) MAF‐23 and b) MAF‐23‐O. Breakthrough curves (filled symbols) and adsorption kinetic curves (open symbols) for c) MAF‐23 and d) MAF‐23‐O using an equimolar C_3_H_6_/C_3_H_8_ (blue/red) mixture at 298 K and 1 bar. Adapted from ref. [83].

**Equilibrium‐based mechanism**. Like ethene/ethane separation, a proportion of propene/propane separation in porous materials are based on π‐complexation between propene and metal ions on different porous materials (porous carbons, zeolites, MOFs, and POFs) and open metal sites in MOFs. In recent years, a number of materials have been explored for propene/propane separation based on π‐complexation and they have displayed efficient propene/propane separation performances: the Ag^I^‐decorated porous polyimide material MPI‐Ag,^[65d]^ MFU‐4L with incorporated Cu^I^ sites,^[78]^ MIL‐101(Cr) loaded with cuprous oxide nanoparticles, Cu^I^‐loaded MIL‐101(Fe), HCPs doped with Ag^I^, aluminosilicates with calcium cations,^[79]^ titanate nanotubes containing Cu^I^,^[80]^ Ag^I^‐doped microporous carbon,^[64b]^ M‐MOF‐74 (M=Co, Mn, and Mg) with high densities of open metal sites,^[81]^ AGTU‐3a with open Ag^I^ sites,^[82]^ and many more. In addition to π‐complexation, some other adsorbents based on the equilibrium mechanism have been developed as well. In 2019, Zhang et al. synthesized the MOF MAF‐23‐O by selective aerobic oxidation of the soft methylene bridges of the organic ligands of MAF‐23 (Figure 12).^[83]^ The oxidation leads to more rigid carbonyl bridges and gives additional guest recognition sites, improving both thermodynamic and kinetic adsorption selectivity. An IAST selectivity of 8 and breakthrough selectivity of 15 for equimolar propene/propane mixtures was achieved for MAF‐23‐O, at 298 K and 1 bar. Recently, by grafting pyrrole onto Cu‐BTC, Li et al synthesized Pyr@Cu‐BTC.^[84]^ Since propene can be preferentially adsorbed to pyrrole by electrostatic interactions, Pyr@Cu‐BTC displayed a high IAST propene/propane selectivity of 8.3 for equimolar propene/propane mixtures, at 298 K and 1 bar. Zhou et al. designed propane‐selective adsorbents by surface tuning and replacing the ligand.^[85]^ They reported a high propane capacity of 8.79 mmol g^−1^ for g‐C_3_N_4_@Zr‐BPDC and a superior propane/propylene selectivity of 1.5 for Zr‐BPYDC at 298 K and 1 bar.

There have also been a few computational screening studies on propene/propane separation. A DFT‐derived force field was applied to describe the adsorption of C_2_–C_3_ olefins and paraffins in CuBTC. This method was then extended to evaluate 94 related Cu‐OMS MOFs for propene/propane separation and 18 MOFs with attractive separation performances were identified.^[86]^ Later, approximately 1 million crystal structures of MOFs, ZIFs, and zeolites were screened by Han et al. for propene/propane separation.^[87]^ GCMC simulations were performed to simulate the selectivity, working capacity, and physical properties of those porous materials. We believe that with the rapid development of computational techniques, HTCS will play a more important role in materials screening for propene/propane separation.

**Kinetic‐based mechanism**. Kinetic‐based separation of propene/propane mixtures makes up a considerable portion of propene/propane adsorption separations. Until now, only a handful of pure silica or high silica zeolites, like Si‐CHA,^[88]^ 4A,^[89]^ ITQ‐12,^[90]^ ITQ‐32,^[91]^ and ZSM‐58,^[92]^ have been reported for the kinetic separation of propene/propane mixtures, while the synthesis of these zeolites is relatively difficult. Decoration/modification has proved to be an effective strategy to improve the separation performance of the traditional zeolites. The directional decoration of the 12‐membered rings of traditional mordenite with ZIF fragments was found to greatly enhance its kinetic propene/propane selectivity.^[93]^ One of the decorated mordenites achieved a high kinetic selectivity of 139 at 298 K, indicating the effectiveness of the decoration strategy.

**Figure 13:**
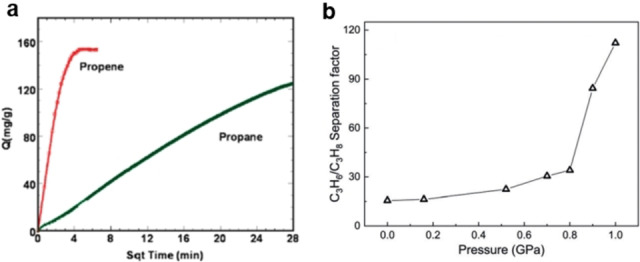
a) Time‐dependent propene and propane adsorption profiles of ZIF‐8 at 303 K and 0.8 bar, as reported in ref. [94a]. b) Evolution of propylene/propane diffusion selectivity as a function of the applied mechanical pressure on ZIF‐8 at 300 K. Adapted from ref. [96].

Over the past decades, a number of MOF materials have been investigated for the kinetic separation of propane and propene.^[94]^ The first and most‐studied example is ZIF‐8, which showed a kinetic selectivity of 125 at 303 K (Figure 13 a).^[94a]^ It is also reported that for ZIF‐67 (ZIF‐8 (Co)), cobalt promotes a more rigid framework and slightly smaller windows than zinc in the isostructural ZIF‐8, and as a result UIF‐67 displays the opposite kinetic propane/propene separation.^[95]^ A quite innovative concept was also proposed in that the pore architecture of the ZIF‐8 material can be mechanically tuned by the application of external pressure up to 1 Gpa, resulting in a significant enhancement in the propylene/propane diffusion selectivity (Figure 13 b).^[96]^

**Figure 14:**
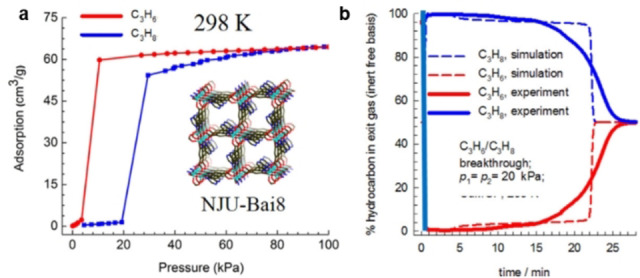
a) C_3_H_6_ and C_3_H_8_ adsorption/desorption isotherms of NJU‐Bai8 at 298 K; insert: structure of DMF‐solvated NJU‐Bai8, representing open pores decorating by pyrimidine rings. b) Experimental and simulated adsorption breakthrough curves of NJU‐Bai8 for C_3_H_6_/C_3_H_8_/He (20 %/20 %/60 %) mixture at 298 K and at a total pressure of 100 kPa. Adapted from ref. [98].

**Gate‐opening effect**. The gate‐opening effect is known for metal–organic frameworks, providing them with advantageous properties gas separation. A few MOFs displaying gate‐opening behavior have been explored for propene/propane separation. The flexible pillared‐layer framework CPL‐1 exhibited a thermo‐responsive gate‐opening behavior towards propene rather than propane, and thus the adsorptive separation of propene over propane was achieved.^[97]^ It is also confirmed that hydrogen bonding plays an important role in the adsorptive separation of propene over propane for CPL‐1. Li et al. also reported that the flexible MOF NJU‐Bai8 can separate propene and propane based on a gate‐opening effect over a wide temperature range from 298 to 348 K (Figure 14).^[98]^ It exhibited a higher propene/propane selectivity at lower pressure (7.2 at 0.05 bar) and exceeded 4 in the range of total pressure up to 100 kPa, at 298 K.

**Figure 15:**
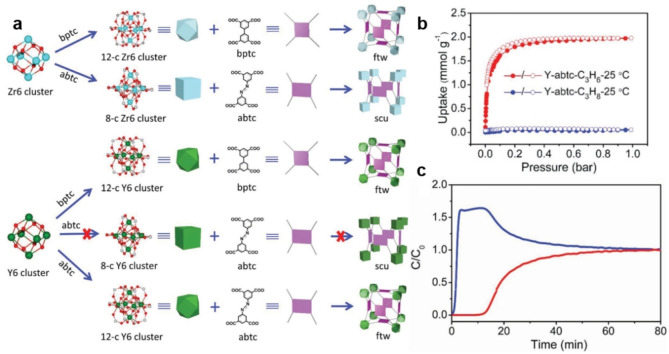
a) Topology analysis of MOFs built from Zr6/Y6 clusters and two tetratopic linkers. b) Single‐component adsorption results of Y‐abtc for propane and propylene at 25 °C. c) Breakthrough curve of Y‐abtc for an equimolar mixture of propane and propylene at 25 °C; red: propylene; blue: propane. Adapted from ref. [99].

**Size exclusion mechanism**. Suitable porosity and precisely controlled channels are required to achieve propene/propane separation via the size exclusion mechanism, which is particularly challenging. Up to now, quite a few MOFs have been reported to achieve the ideal propene/propane separation by the molecular sieving effect. By controlling the surface chemistry and pore size (usually substitutions of the organic ligand and inorganic nodes), the MOF structures can be finetuned, thereby achieving ideal molecular sieving properties for gas separations. The ultra‐microporous fluorinated MOF NbOFFIVE‐1‐Ni (also referred to as KAUST‐7), which can fully sieve propane from propene/propane mixtures under ambient conditions, was reported.^[23]^ The sieving effect is attributed to the selected bulkier (NbOF_5_)^2−^ hindering the rotation of the pyrazine moieties, and thus dictating the pore aperture size and its maximum opening. However, due to the flexible aperture, the effect can only be achieved at low pressures. Another MOF material, Y‐abtc (with abtc=3,3′,5,5′‐azobenzene‐tetracarboxylates) with cage‐like pores, was prepared by a topology‐guided design strategy; it could adsorb propene, but completely excluded propane (Figure 15).^[99]^ Precise tuning of the pore aperture and optimal pore dimensions for propene/propane separation was achieved by replacing secondary building units and through the judicious selection of structure topology, inorganic nodes, and organic linkers. Table 5 provides an overview of selected state‐of‐the‐art porous materials for C_3_H_6_/C_3_H_8_ separation.

**Table 5:** Overview of selected state‐of‐the‐art porous materials for C_3_H_6_/C_3_H_8_ separation. Sel.: selectivity.

Material	Sel.	*T* [K]	*P* [bar]	C_3_H_6_ uptake [mmol g^−1^]	Ref.
MPI‐Ag	7.2	298	1	1.07	[65d]
15 %Cu@MIL‐101(Cr)	12.2	303	1	3.5	[79a]
12 %Cu@MIL‐101(Cr)	9.5	303	1	4.4	[79a]
Cu(0.6)@MIL‐100(Fe)	13.2	323	1	3.3	[79b]
SAM‐HCP‐Ag‐3	24	298	1	1.75	[79c]
MC‐S‐Ag‐3	2.4	298	1	5.5	[64b]
Co‐MOF‐74	46	298	1	7.3	[81]
AGTU‐3a	7	298	1	1.22	[82]
MAF‐23‐O	9	298	1	≈1.35	[83]
Pyr1/3@Cu‐BTC	8.3	298	1	7.6	[84]
NJU‐Bai8	4.2	298	1	2.89	[98]
Zr‐BPYDC^[a]^	≈1.5	298	1	≈8.4	[85]

[a] Zr‐BPYDC is propane‐selective. The selectivity is C_3_H_8_/C_3_H_6_ selectivity and uptake is C_3_H_8_ uptake.

## 5 Separation of C_8_ Aromatics

The mixture at the top of the xylene splitter typically contains about 19 % ethylbenzene, 44 % *m*‐xylene, 20 % *o*‐xylene, and 17 % *p*‐xylene, and must be separated into the individual isomers for specific end‐use, as mentioned before.^[100]^ The separation of xylene isomers is highly challenging as a result of the similar physicochemical properties of these isomers (Table 1). In particular, the extremely close boiling points makes it impracticable to separate them efficiently by distillation, because of the large number of theoretical plates required.^[101]^ The adsorptive strategy is the main technology for industrial separation of xylene isomers, of which about 60 % PX separation is performed by selective adsorption with zeolites. Industrially, separation of PX is mainly performed on large‐scale simulated moving bed (SMB) units, which has been implemented in three industrial‐scale processes (i.e., UOP′s Parex, Toray's Aromax, and IFP′s Eluxyl).^[102]^ The Parex process, first commercialized by UOP for the production of PX in 1971, was pioneering in applying the principle of adsorption separation on an industrial scale. Later, in the early 1970s, the Aromax process was developed by Toray Industries, and in 1994, IFP commercialized the Eluxyl adsorption process. In refineries, the amount of PX in xylene mixtures varies from 17 to 24 wt %. By these technologies, a liquid mixture can be separated by SMBs around 453 K and 9 bar, achieving the isolation of PX with a purity grade superior to 99 %.^[103]^ The adsorbents employed in SMBs are FAU‐type zeolites X and Y, ion‐exchanged with, for example, Na^+^, K^+^, and Ba^2+^.^[104]^ Besides the FAU zeolite framework structure, other zeolite framework structures, like MFI^[105]^ and MOR,^[106]^ also have been tested for PX separation. The dominant role adsorbents play in these separation process makes it necessary to explore and develop highly efficient adsorbents. Advanced porous materials, like MOFs and COFs, have been extensively explored for separation of xylene isomers.^[107]^ However, for zeolite‐based separation materials not much progress has been made in recent years, although it should be clear that their durability and thermal stability offer many prospects (mainly in industrial applications) over the more recently explored MOFs and POFs.

**Figure 16:**
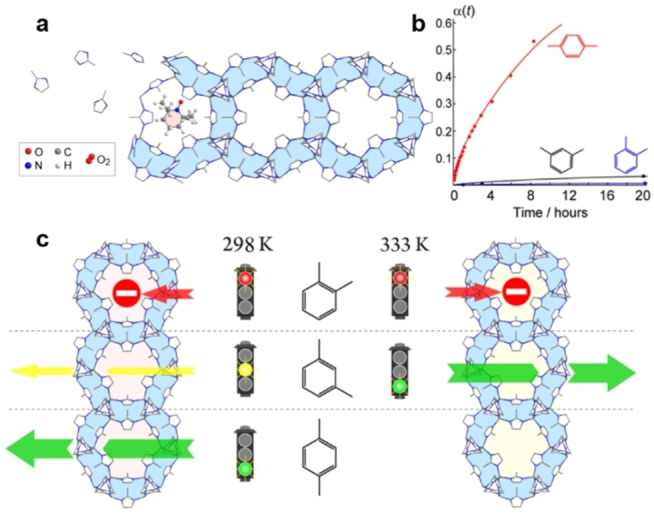
a) Schematic of the self‐assembly of TEMPO@ZIF‐8. b) Kinetic curves *α*(*t*) for TEMPO@ZIF‐8 with different xylenes (indicated) at room temperature. c) Schematic of the separation of xylene isomers in TEMPO@ZIF‐8. Adapted from ref. [108].

**Figure 17:**
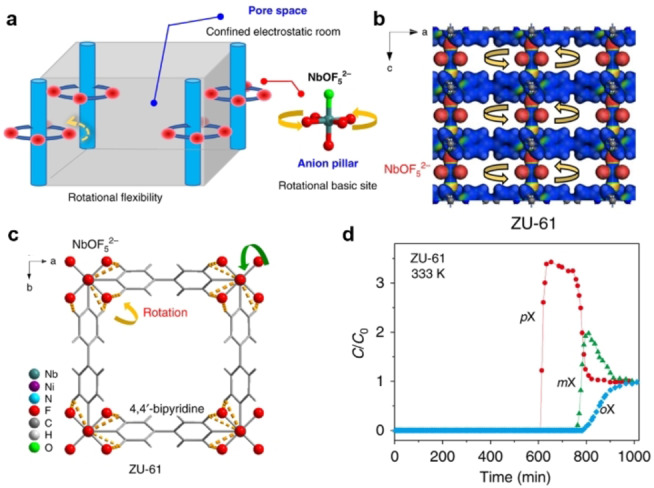
a) Schematic illustration of the porous adsorbent with Lewis‐basic binding sites and rotational flexibility. b, c) Structure of ZU‐61 with the rotational ligand NbOF_5_^2−^ anion and bipyridine. d) Breakthrough curves for 1:1:1 PX/MX/OX separations with ZU‐61 at 333 K. Adapted from ref. [112].

**Figure 18:**
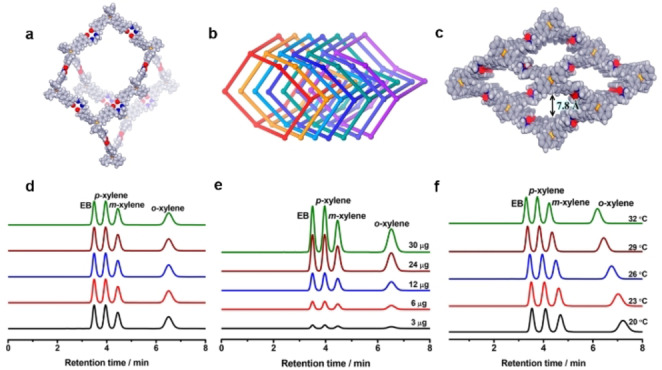
a) Structural representations of salen‐COFs. A space‐filling model of an adamantine‐like cage in salen‐COFs. b) Interpenetration of seven diamondoid nets in COFs. c) A space‐filling model of the 3D structure of salen‐COFs viewed along the *a*‐axis. HPLC chromatograms of EB and xylene isomers on the salen‐COF 1 packed column d) for five replicate experiments, e) with different injected masses, and f) at 20–32 °C. C gray; N blue; H white; O red; the central C/Si in C/Si‐THBA is yellow. Adapted from ref. [114].

Polyukhov et al. synthesized ZIF‐8 with the stable nitroxide TEMPO ((2,2,6,6‐tetramethylpiperidin‐1‐yl)oxyl) permanently entrapped in the pores (Figure 16).^[108]^ The diffusion of xylene isomer molecules in the ZIF‐8 cavities could be significantly modulated by changing the temperature within the range 298–333 K, as the window size of the TEMPO@ZIF‐8 changes with temperature. In TEMPO@ZIF‐8, PX can be easily separated from OX and MX with MX/OX separation efficiencies up to 93–95 % at 298 K. MOFs also can be used as a stationary phase for the chromatographic separation of xylene isomers and ethylbenzene.^[109]^ Functionalized Zr‐BTB nanosheets with an untwisted stacking mode were examined for chromatographic separation.^[110]^ The stacking was untwisted and ordered sub‐nanometer pores were created by preheating; a column coated with the untwisted nanosheets exhibited practical PX selectivity from xylene isomers. In 2018, Long et al. reported two MOFs, Co_2_(dobdc) and Co_2_(m‐dobdc), with unsaturated cobalt(II) sites for the separation of C_8_ aromatic isomers; their separation performances depended on differences in interactions between each isomer and the two open cobalt(II) centers.^[35c]^ All four isomers can interact with both the unsaturated cobalt(II) sites and the linker aromatic rings in Co_2_(dobdc) through arene π–π interactions with the dobdc^4−^ linker. Owing to the different strengths of its binding affinities to those isomers (OX> EB> MX> PX), Co_2_(dobdc) can separate all four isomers effectively. In contrast, Co_2_(m‐dobdc) has similar binding to MX and EB and can thus distinguish only three of the four isomers. This indicates that the subtle structural differences between Co_2_(dobdc) and Co_2_(m‐dobdc) have a huge impact on their adsorption properties. Recently, by refining the pore size at sub‐Angstrom resolution (7.4–6.3 Å in steps of 0.2 Å), Schröder et al. reported a series of MFM‐300(M) (M=In, V, Fe, Al) for the separation of xylene isomers at room temperature, achieving selectivity (PX< OX< MX) and separation factors of 4.6–18 for PX and MX.^[111]^ Xing al. also reported a ZU‐61 MOF with accessible and rotational Lewis basic sites for the adaptive molecular discrimination of xylene isomers (Figure 17).^[112]^ Through the rotation of fluorine atoms, the anionic sites can adapt to the shape specific isomers, therefore enabling ZU‐61 to efficiently separate xylene isomers. ZU‐61 exhibited a preferential adsorption sequence of OX> MX >PX and both high MX uptake capacity (3.4 mmol g^−1^, 7.1 mbar) and MX/PX separation selectivity (2.9, obtained from breakthrough curves) were achieved at 333 K. The HTCS method has also been used to investigate the separation of xylene isomers. Sholl et al. performed GCMC simulations for ≈4700 MOF structures from the Computation‐Ready, Experimental MOF database to identify PX‐ selective MOFs.^[113]^ The two best‐performing MOFs (MIL‐140B and MOF‐48) were synthesized and evaluated by breakthrough experiments and modeling. The PX selectivities of MIL‐140B and MOF‐48 are lower than the simulated results but exceeded that of zeolite BaX. The diversity, high stability, and adsorption capacity of POFs also endow them with a number of benefits for the separation of xylene isomers. Host–guest interactions between POFs and xylene isomers can by tuned by choosing suitable external crosslinkers and building blocks. The triptycene‐like microporous organic polymer POP‐1 was reported for the separation of xylene isomers.^[109c]^ The weak CH/π interactions were successfully used to tune the host–guest interactions and to achieve separation of xylene isomers. In 2019, COFs were first reported by Huang and co‐workers for the separation of xylene isomers and ethylbenzene.^[114]^ Two pairs of microporous 3D salen‐ and Zn(salen)‐based COFs served as the stationary phase and were examined for the chromatographic separation of C_8_ aromatics. All four COFs present a sevenfold interpenetrated diamondoid open framework with wide tubular channels of about 7.8 Å decorated with salen or Zn (salen) units (Figure 18 a–c). C_8_ aromatic molecules stack in the COFs by an edge‐to‐face configuration. Only the two methyl groups of OX can interact with the polar salen groups of the four COFs, and the hydrogen bond lengths between OX and COFs are shorter than with other isomer molecules. One salen‐COF exhibited excellent column efficiency and precision. The retention time remained the same for EB and xylene isomers when the injected masses increased from 3 to 30 μg, and only a slight decrease in selectivity was observed as temperature increased from 20 to 32 °C (Figure 18 d–f). Table 6 provides an overview of selected state‐of‐the‐art porous materials for separation of xylene isomers.

**Table 6:** Overview of selected state‐of‐the‐art porous materials for separation of xylene isomers.

Material	Selectivity			*T* [K]	Uptake [mmol g^−1^]	Ref.
*PX preference*	*PX/MX*	*PX/OX*	*PX/EB*		*PX*	
Nano‐BaX	7.2	2.8	3.8	423	0.97	[104i]
Nano‐KX	5.4	2.4	3.2	423	0.95	[104j]
H/ZSM‐5	25.0	16.8	6.8	403–443	1.34	[105c]
BaY	3.8	3.9	1.5	453	–	[106]
DUT‐8(Cu)	7.2	5.4	5.9	298	1.8	[107a]
MIL‐125(Ti)‐NH_2_	3.0	2.2	1.6	298	1.2	[107b]
Cu(CDC)	7.0	10.0	5.0	298	1.1	[107c]
						
*OX preference*	*OX/MX*	*OX/PX*	*OX/EB*		*OX*	
Co_2_(dobdc)	2.5	3.9	1.21	423	3.6	[35c]
COF 1	2.0	–	–	293–305	–	[114]
MIL‐53(AI)	5.1	5.2	8.2	323	3	[107e]
						
*MX preference*	*MX/PX*	*MX/OX*	*OX/PX*		*MX*	
MFM‐300(In)	4.6	2.9	1.6	293	≈3.6^[a]^	[111]
MFM‐300(V)	15	3	5	293	3.88^[a]^	[111]
MFM‐300(Fe)	18	6	3	293	–	[111]
ZU‐61^[a]^	2.9	–	–	333	3.4	[112]
POP‐1	2.0	2.3	0.89	298	2.74	[109c]

[a] Uptake measured at 318 K.

## 6 Concluding Remarks, Challenges, and Outlook

**Figure 19:**
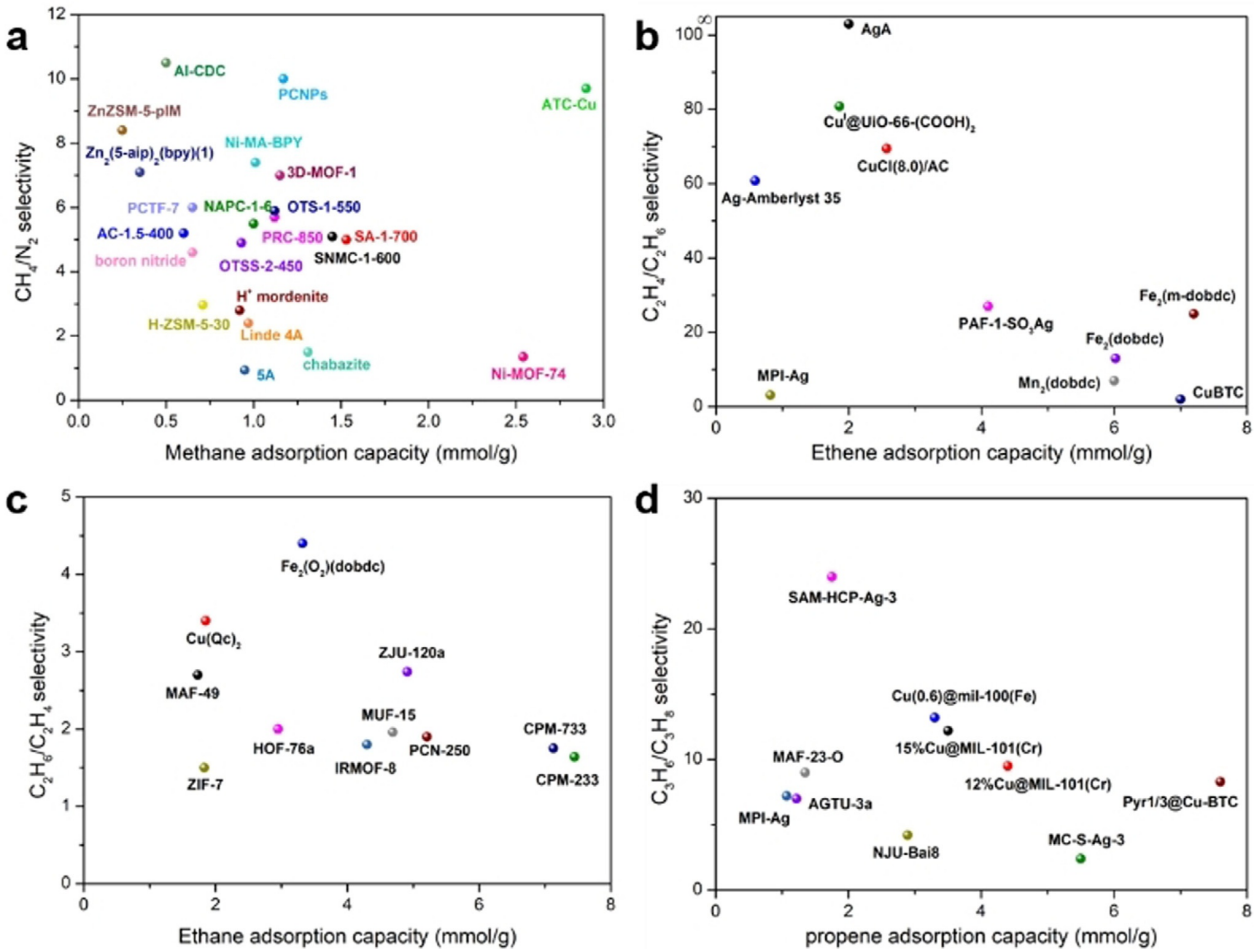
Comparison of a) CH_4_/N_2_ adsorption selectivity and volumetric CH_4_ uptake, b) C_2_H_4_/C_2_H_6_ adsorption selectivity and volumetric C_2_H_4_ uptake, c) C_2_H_6_/C_2_H_4_ adsorption selectivity and volumetric C_2_H_6_ uptake and d) C_3_H_6_/C_3_H_8_ adsorption selectivity and volumetric C_3_H_6_ uptake in typical porous materials.

The past three decades have witnessed the fast growth and development of adsorptive purification and separation of hydrocarbons with a wide range of porous materials. We have summarized and discussed the recent developments of porous materials research for the separation of methane/nitrogen, ethene/ethane, propene/propane, and C_8_ aromatics mixtures. Great progress has been made in adsorptive separation of hydrocarbons and the research on a wide variety of porous materials with different compositions and structures is going at full blast. Figure 19 provides a summary of the reported selectivities and volumetric uptakes of typical porous materials for the separation of methane/nitrogen, ethene/ethane, and propene/propane. Traditional materials, like zeolites, have played and will continue to play an important role in gas separations. Since several zeolites have been successfully applied in industrial processes, zeolites have obvious advantages for practical application of hydrocarbon separations. Modifications of zeolites and the synthesis of new zeolite materials with novel framework structures and chemical compositions are the two strategies for the development of new, better performing zeolite‐based adsorbents. In contrast, for another traditional material, porous carbons and activated carbons, the irregular structures as well as the difficulty in tuning pore shapes and sizes greatly limit their practical application and separation performance. Advanced porous materials, including MOFs and POFs, exhibit great potential and appear quite promising for the purification and separation of hydrocarbons (Figure 19). They are currently being researched in great detail. The precise control of both their surface chemistry and aperture size has proven to be a successful strategy, offering some MOF and POF materials extraordinary separation selectivities.

Although progress has been made, the separation of hydrocarbons using porous materials still faces many challenges for future successful applications. These challenges can be summarized as follows:

1. One of the greatest challenges involves developing adsorbents that perform well in terms of both selectivity and adsorption capacity. Although MOFs and POFs are promising, attention should still be paid to traditional materials. Zeolites, MOFs, and POFs are all promising candidates; however, for zeolites, especially for modified zeolites, their adsorption capacities need to be improved. Many MOFs and POFs possess either high adsorption capacities or high selectivities. Studying the structural and chemical properties of porous materials and understanding the influence of slight differences in their physicochemical properties should be further evaluated. Furthermore, emphasis should also be put on exploiting new MOFs and POFs and industrializing the best ones in practical applications, which also require the proper shaping of these porous materials. The combination of traditional and advanced porous materials also would be an effective strategy for the preparation of novel adsorbents.
2. Developing ethane‐selective and propane‐selective adsorbents is still difficult. More emphasis should be put on the development of highly efficient ethane‐selective and propane‐selective adsorbents for light olefin separation systems. For xylene isomers, besides improvement in the selectivity of PX‐selective adsorbents, the development of MX‐adsorption materials for the removal of MX in PX‐containing gas streams is also challenging but appealing.
3. Compared with the more classic use of single‐component gas adsorption isotherms, the dynamic breakthrough experiment has now become a basic and common evaluation method for porous adsorbents. In addition to developing novel and efficient porous materials, in the future, more properties of adsorbents, especially those related to industrial applications, including stability, mechanical properties, shaping, and regeneration, should be used as the criteria for the evaluation of adsorbents.
4. Advances in computational methods have already assisted in elucidating adsorption and separation mechanisms, including predicting the structure of materials and guiding the design of new adsorbents. High‐throughput computational screening can play a role in accelerating the design and development of highly efficient adsorbents. However, there is a gap between experimental synthesis methods and computational methods. For example, the proposed hypothetical structures are often difficult or impossible to synthesize experimentally. Computational works should take a thorough consideration of both experimentally synthesized structures and hypothetical framework structures. With the rapid development of computer science, computational screening methods, including machine learning, will continue to play a significant role in boosting the design, development, and application of porous materials in hydrocarbon separation and purification.

Despite many potential challenges, there is no doubt that important improvements will be achieved in the field of adsorptive separation and purification of hydrocarbons. We can expect that in the near future, more efforts will be devoted to the implementation of porous materials, MOFs in particular, in large‐scale industrial applications, thereby focusing on improving the stability in the presence of gas‐phase impurities and reducing the overall cost of adsorbents as well as process design.

## Conflict of interest

The authors declare no conflict of interest.

## Biographical Information

*Yaqi Wu is currently a postdoctoral researcher in Prof. Weckhuysen's group at Utrecht University. She received her PhD degree in Industrial Catalysis from the Dalian Institute of Chemical Physics, Chinese Academy of Sciences (2020) under the direction of Prof. Yunpeng Xu and Prof. Zhongmin Liu. Her current research interests include developing MOF thin films for ethene polymerization and gas adsorption*.

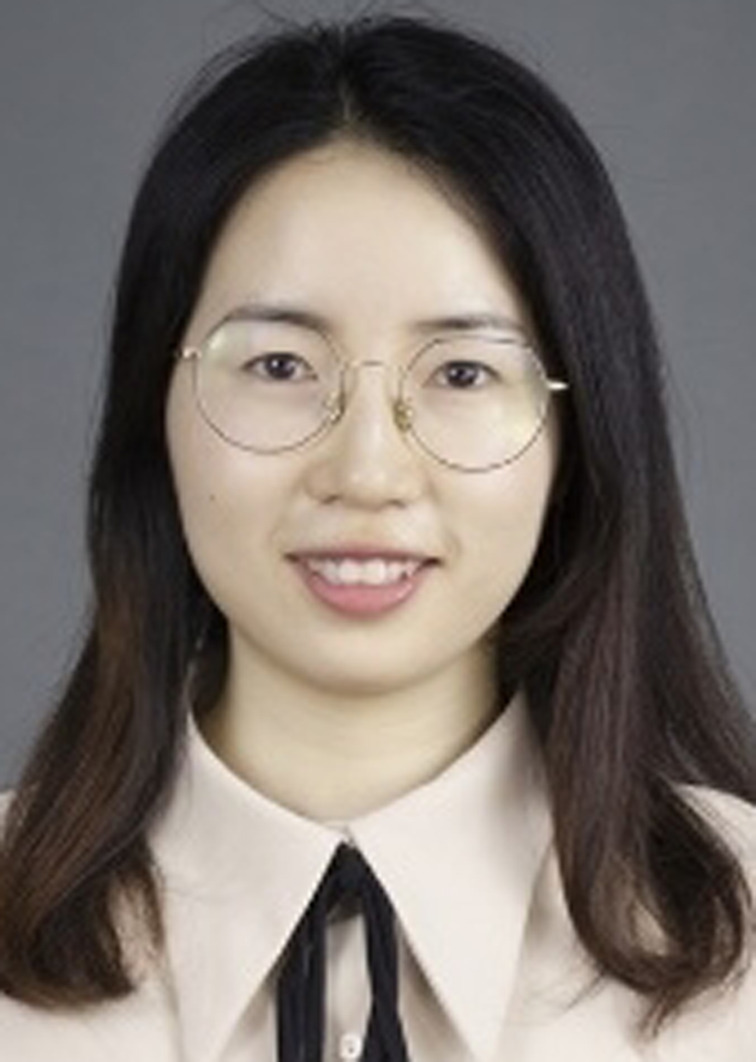

## Biographical Information

*Bert M. Weckhuysen obtained his PhD degree from K. U. Leuven (Belgium) in 1995. After postdoctoral stays at Lehigh University (PA, USA) and Texas A&M University (TX, USA), he became full Professor at Utrecht University (The Netherlands) in 2000. His research focuses on the development and use of in situ and operando spectroscopy for studying solid catalysts under realistic reaction conditions at different length scales*.

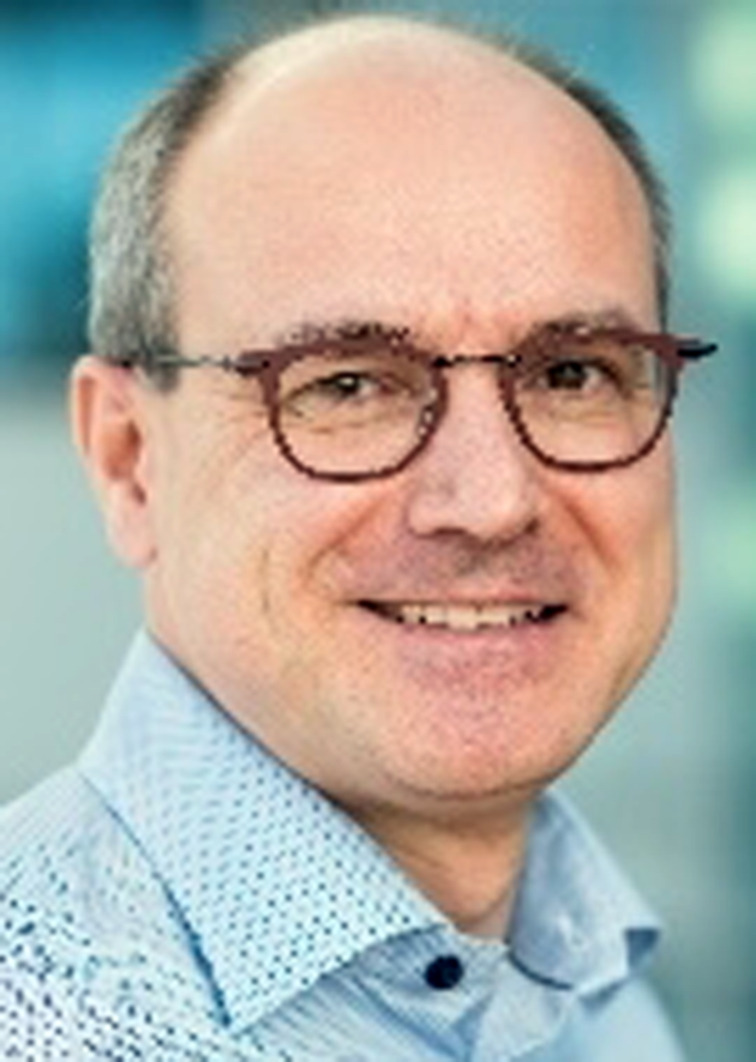
